# Supplementary material for: Tuning the interactions between chiral plasmonic films and living cells
Source: Nat Commun. 2017 Dec 8;8:2007. doi: 10.1038/s41467-017-02268-8 (PMC5722823; doi:10.1038/s41467-017-02268-8)
Supplement: Supplementary file 1 — Supplementary Information [file 41467_2017_2268_MOESM1_ESM.pdf]

Supplementary Methods

Materials

All reagents were purchased from Sigma-Aldrich, unless otherwise stated. All chemicals were used as received. Deionized (DI) water from a Milli-Q device (18.2 MΩ; Millipore, Molsheim, France) was used throughout this study. RPMI-1640 and Dulbecco's phosphate-buffered saline (DPBS; no calcium, no magnesium) were purchased from Gibco™. 4′, 6-Diamidino-2-phenylindole (DAPI) was purchased from Thermo Scientific. ActinRed™ 555 ReadyProbes® Reagent and the LIVE/DEAD® Cell Imaging Kit were purchased from Molecular Probes™. The anti-vinculin antibody (ABfinity rabbit monoclonal antibody) and the goat anti-rabbit IgG (H+L) secondary antibody (conjugated with Alexa Fluor 488) were purchased from Invitrogen. The Cell Counting Kit-8 was purchased from Beyotime. The NG108-15 cell line was obtained from the China Center for Type Culture Collection (Wuhan, P.R. China). RIPA lysis buffer IV, anti-GAPDH antibody, anti-MYCN antibody and HRP-conjugated Goat anti-Rabbit IgG, western blot kit was purchased from Sangon Biotech (Shanghai) Co., Ltd. L-penicillamine and D-penicillamine (denoted as L-Pen and D-Pen, respectively) were purchased from Sigma-Aldrich.

Methods

All glassware was cleaned with freshly prepared *aqua regia* and rinsed thoroughly with DI H_2O before use. TEM images were acquired with a Jeol JEM-2100 transmission electron microscope (operated at an acceleration voltage of 200 kV). SEM images were acquired with a Hitachi S-4700 scanning electron microscope. All UV–Vis results were acquired with a UNICO 2100 PC UV–Vis spectrophotometer and were processed with the Origin Lab software. CD spectra were obtained with a Chirascan CD spectrometer from Applied Photophysics Limited. Confocal imaging

was performed with a Leica TCS SP8 confocal fluorescence microscope.

Synthesis of Au NPs[1]

Au NPs were synthesized with a seed-mediated growth method. Seeds (Au NPs of 13 ± 1 nm) were synthesized with a routine method. Trisodium citrate (2 mL, 38.8 mM) was quickly added to a boiling solution of $HAuCl_4$ (40 mL, 0.5 mM) with vigorous stirring and refluxed until there was no further color change in the solution. Aqueous trisodium citrate solution (0.8 mL, 1% by weight, freshly prepared) and 13 nm Au NP seeds (4 mL) were then quickly added to a boiling aqueous solution of $HAuCl_4$ (200 mL, 0.25mM) with vigorous stirring and refluxed. After several minutes, the color of the solution changed from pale yellow to brilliant red. After boiling for 10 min, the heat source was removed to allow the reaction solution to cool to room temperature.

Modification of Au NPs

Au NPs (50 mL) were centrifuged (3500 rpm, 6 min), and suspended in 50 mL of Milli-Q water. L- or D-Pen (1 M) was added to the Au solution to a final concentration of 50 mM, and incubated in a 60 °C water bath for 2 h.

Preparation of monolayer chiral Au films[2]

L/D-Pen functionalized Au NPs were centrifuged (3500 rpm, 6 min) and suspended in 10 mL of Milli-Q water in a beaker; and 3 mL of *n*-hexane was slowly added to the beaker. When 10 mL of alcohol was added to the beaker, a monolayer of Au film appeared at the oil–water interface. When the n-hexane was evaporated completely,

the Au film was transferred to a glass surface or PDMS. Before use, the glass substrates were cleaned with piranha solution for 2 h at 60 °C.

Preparation of PDMS

The fabrication of PDMS film is described below: The PDMS mixture of base and cross-linker (Dow Corning Sylgard 184; the weight ratio of base to cross linker was 10:1) was stirred at least for 20 min, degased in vacuum for 10 min to get rid of bubbles on a clean glass at room temperature, solidified at 70 °C for 2 h, and carefully peeled off from the glass to get PDMS thin film. It was then immersed in APTES ((3-aminopropyl) triethoxysilane) solution (30 μL APTES in 30 ml ethanol) for 1 h, then washed with ethanol and dried in the oven at 60°C for 1 h.

Preparation of L- or D-Pen-PDMS

The surface group of PDMS was amino group. To prepare L- or D-Pen-PDMS, 50 mM L- or D-penicillamine dissolved in phosphate buffer containing 0.5 mM 1-(3-dimethyl-aminopropyl)-3-ethylcarbodiimide hydrochloride (> 98%) and 0.05 mM N-hydroxysuccinimide (> 98%) to activate them. 6 h later, amino PDMS was put in the mixture solution, and the solution was incubated at 60 °C with shaking for 4 h. After 4 h, the modified PDMS was washed with phosphate buffer for 3 times to remove the physically adsorbed penicillamine enantiomer.

Preparation of L- or D-Pen-Au coating

PDMS was sputtered with gold (Au coating) using a sputter coater. After that, the Au coatings were incubated in L- or D-Pen solution with a concentration of 50 mM at a 60 °C water bath for 2 h. After that, the Au coatings were rinsed with phosphate buffer for 3 times to remove the physically adsorbed penicillamine. And, we named the Au coatings modified with L- or D-Pen enantiomer as the L- or D-Pen-Au coating, respectively.

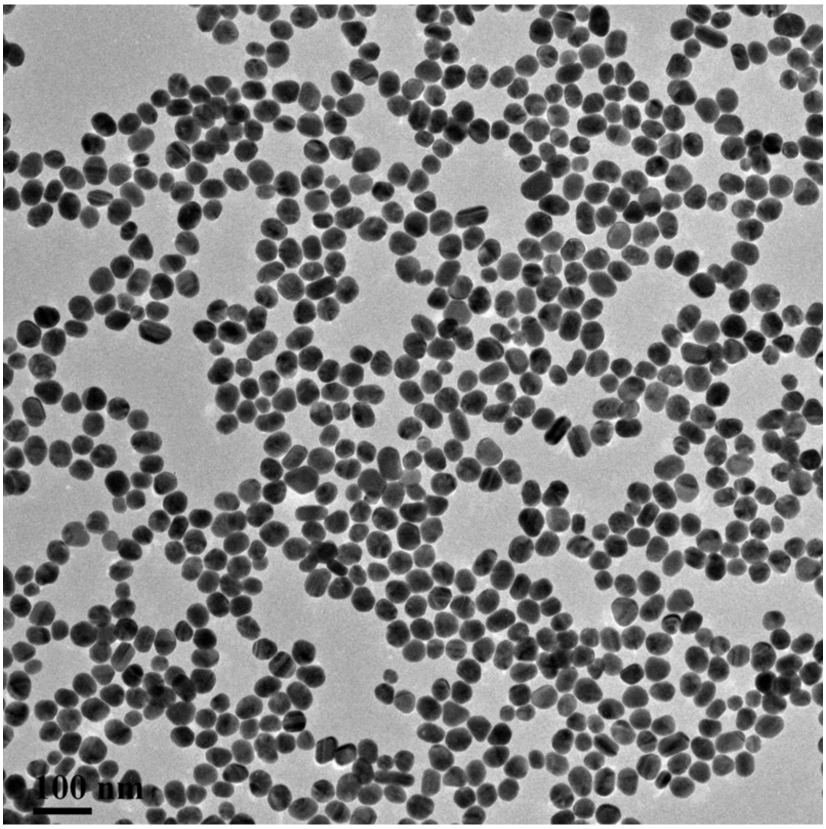

Supplementary Figure1 | TEM image of 47 ± 5 nm Au NP.

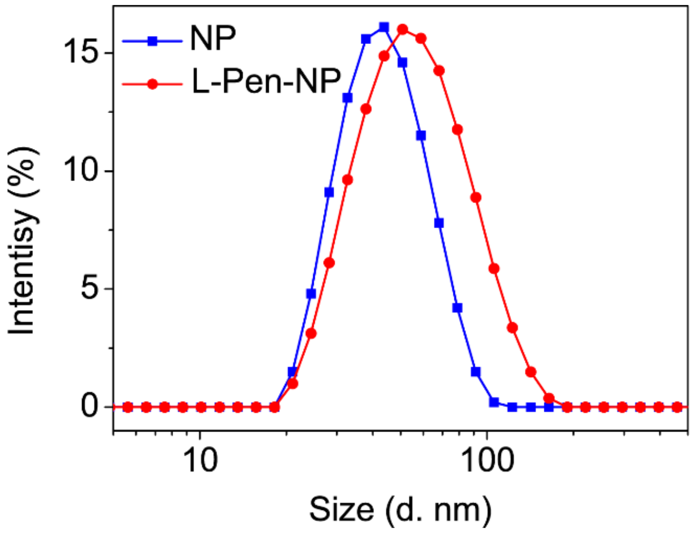

Supplementary Figure2 | Dynamic light scattering (DLS) size of individual NPs and L-Pen molecules modified NPs.

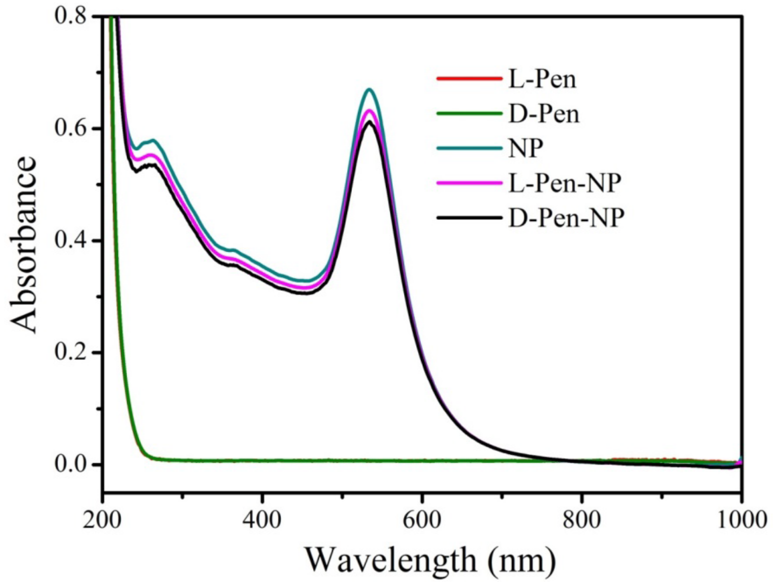

Supplementary Figure 3 | UV-Vis spectra of L/D-Pen and L/D-Pen modified Au NP.

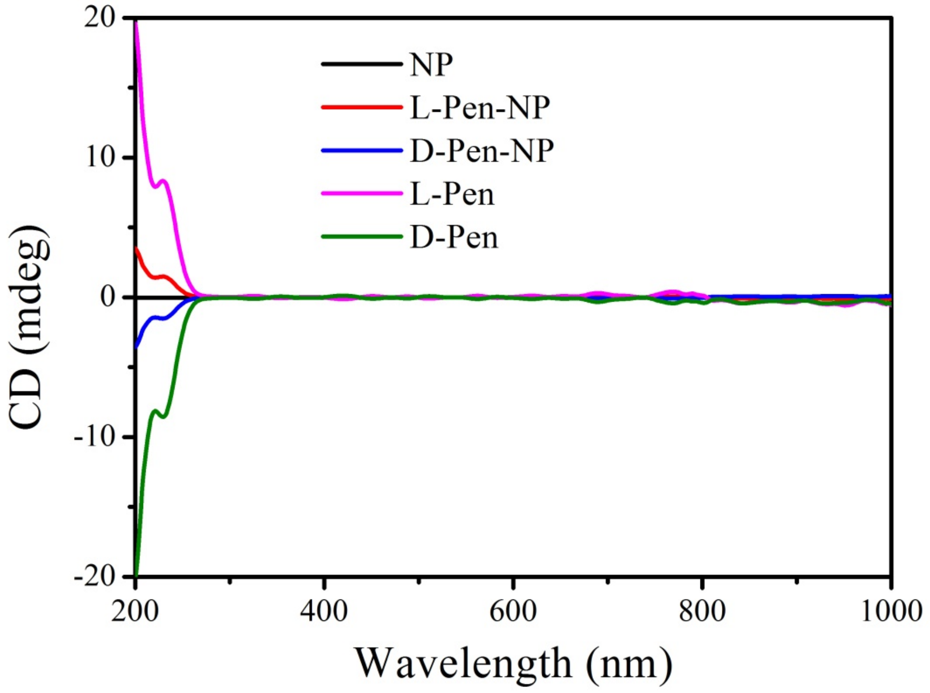

Supplementary Figure 4 | CD spectra of L/D-Pen and L or D-Pen modified Au NP.

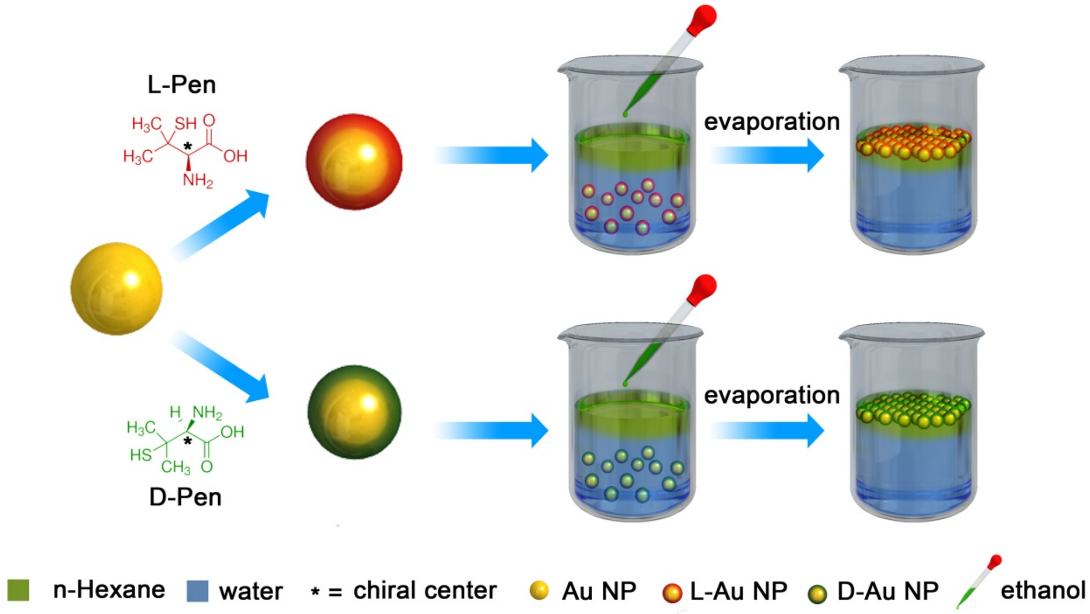

Supplementary Figure 5 | Illustration of preparation of Au NP film.

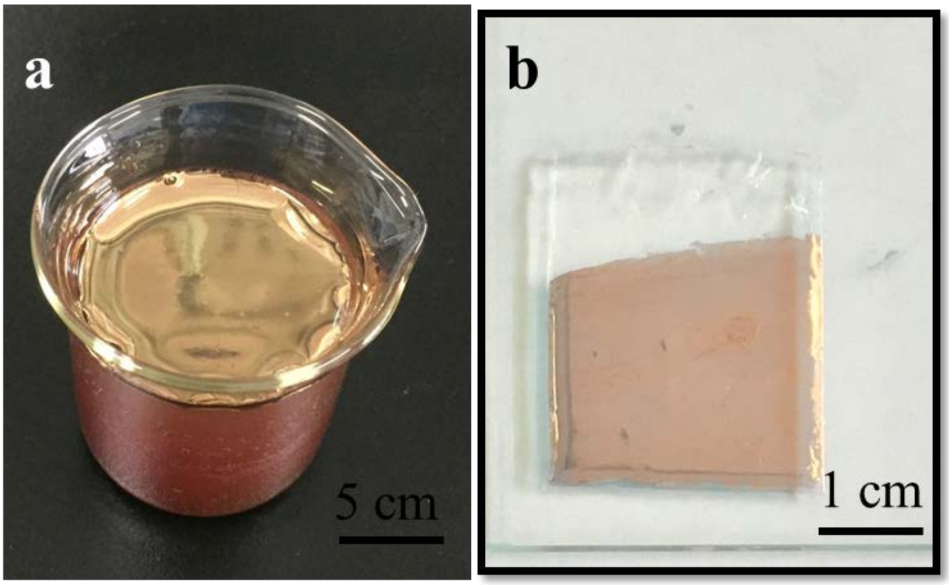

Supplementary Figure 6 | a) Au NP film on the interface of water and n-hexane and b) transfered to PDMS.

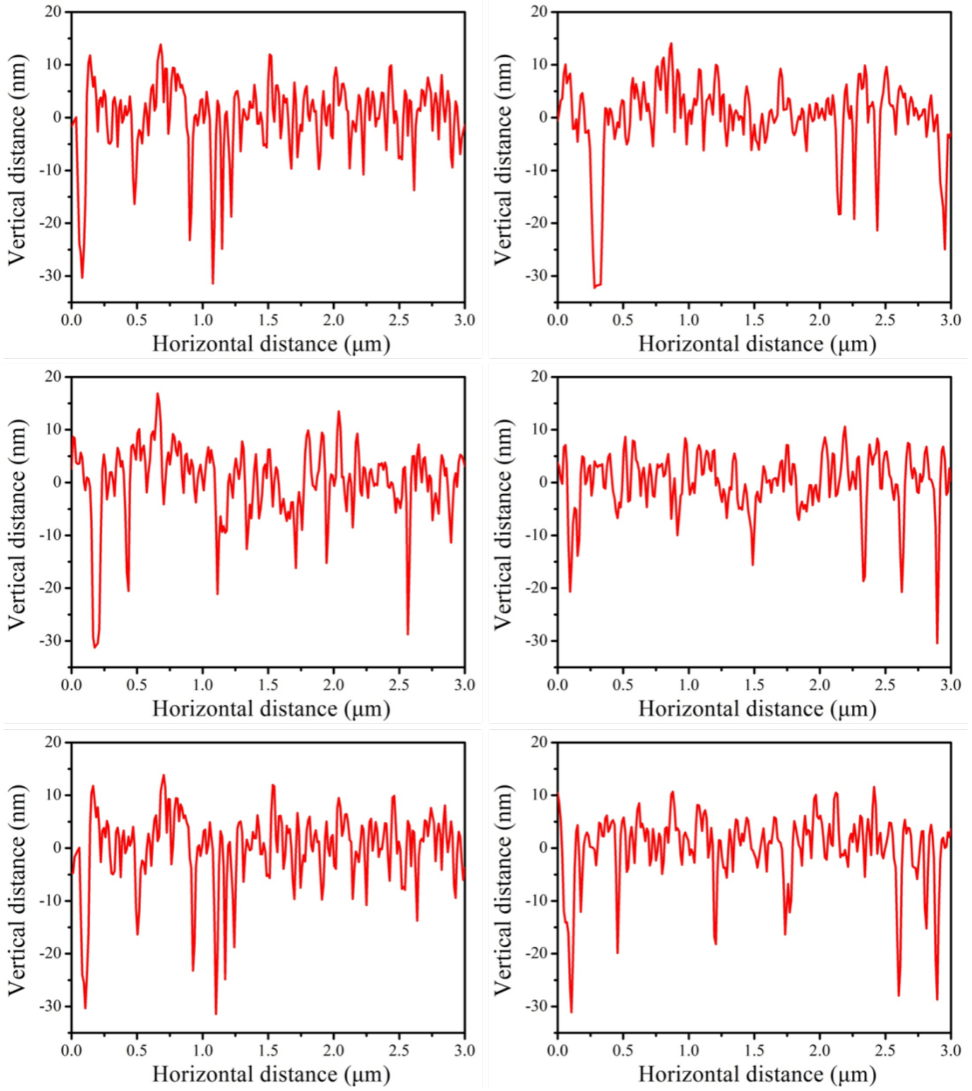

Supplementary Figure 7 | Vertical distance of Au NP film (six replicates of the same experiment).

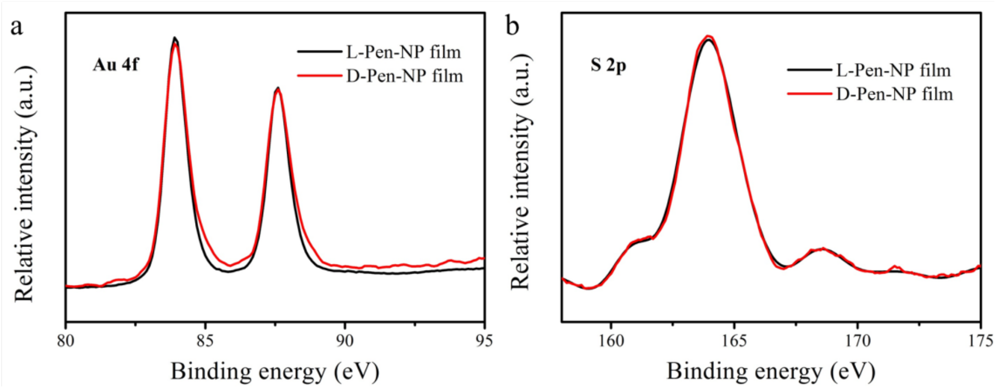

Supplementary Figure 8 | X-ray photoelectron spectroscopy of (a) Au and (b) S element of the L/D-Pen modified Au NP films.

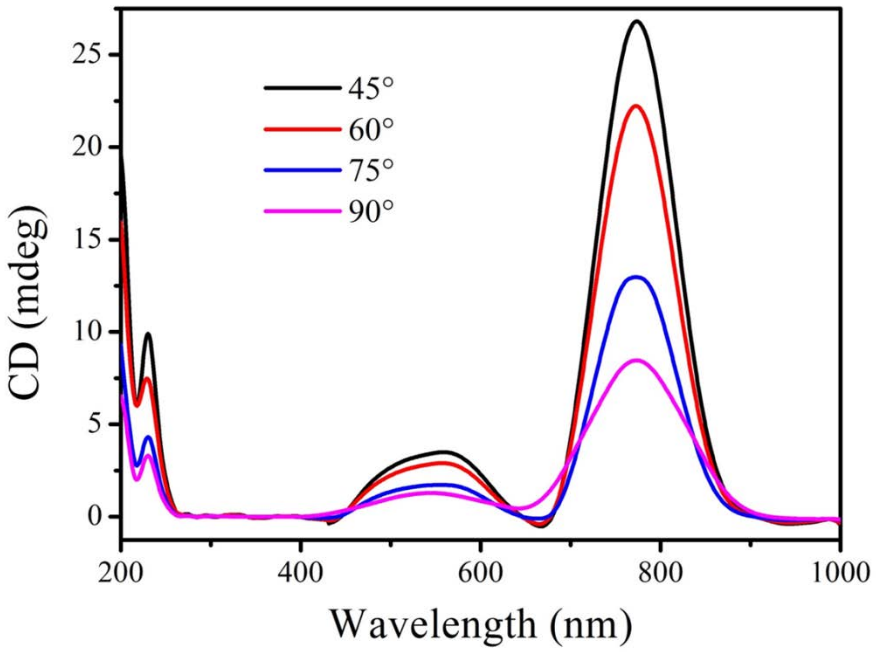

Supplementary Figure 9 | CD spectra of L-Pen-NP film displaying different angle with light.

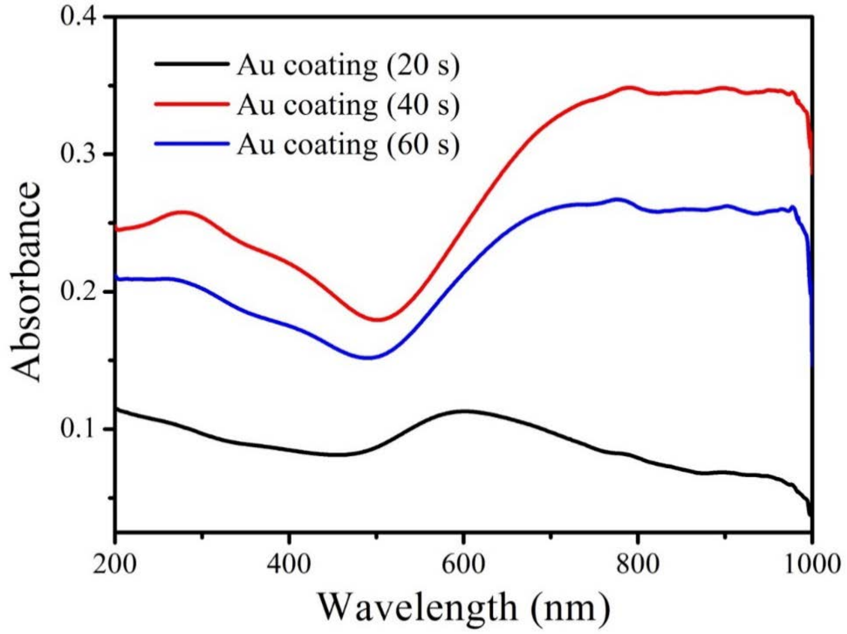

Supplementary Figure 10 | UV-Vis spectra of Au coating (PDMS sputtered by gold on a SBC-12 sputter coater) with different sputtering time (10 mA for 20 s, 40 s, 60 s).

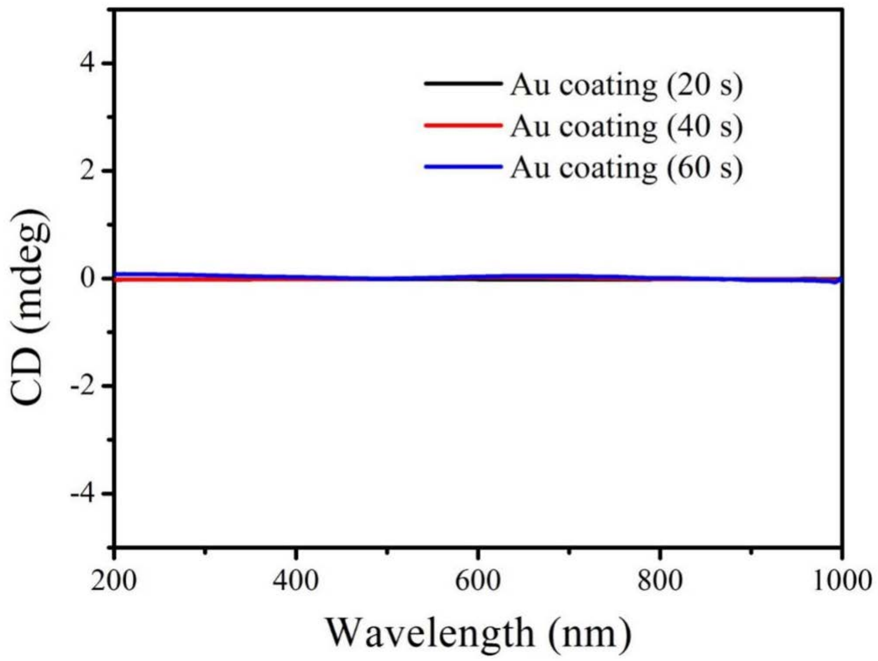

Supplementary Figure 11 | CD spectra of Au coating (PDMS sputtered by gold on a SBC-12 sputter coater) with different sputtering time (10 mA for 20 s, 40 s, 60 s).

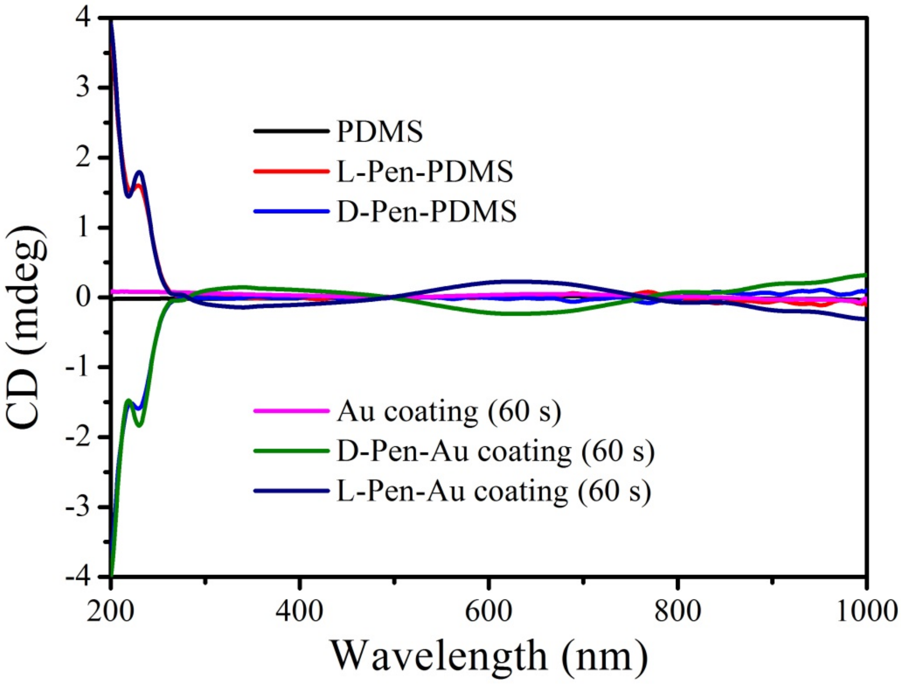

Supplementary Figure12 | CD spectra of PDMS, Au coating (60 s) and L- or D-Pen modified PDMS and Au coating (60 s).

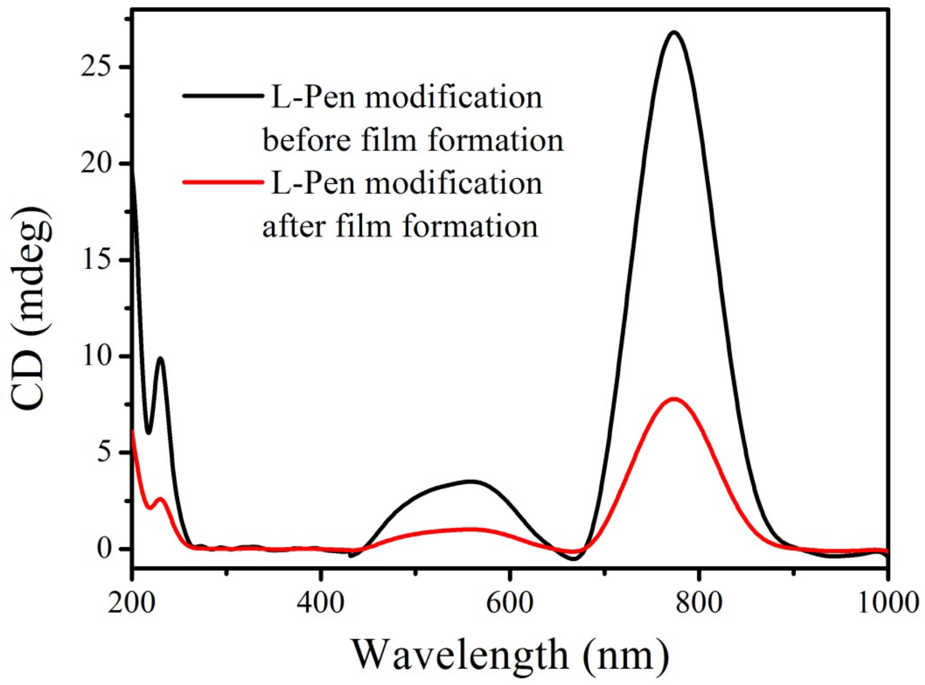

Supplementary Figure 13 | CD spectra of chiral Au NP film modified with L-Pen before and after film formation.

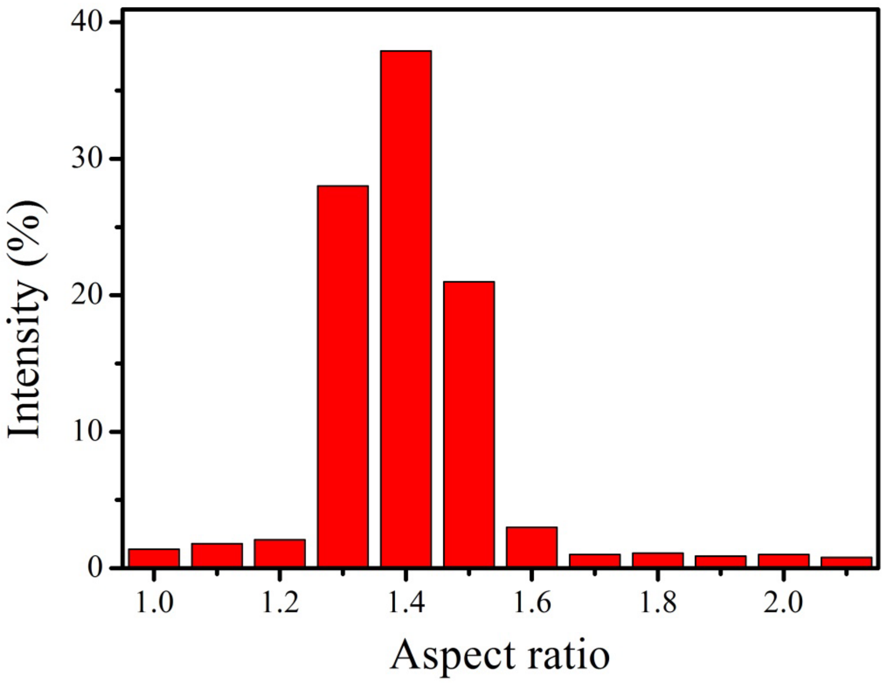

Supplementary Figure14 | The aspect ratios of NPs determined by bright-field TEM for Au NPs.

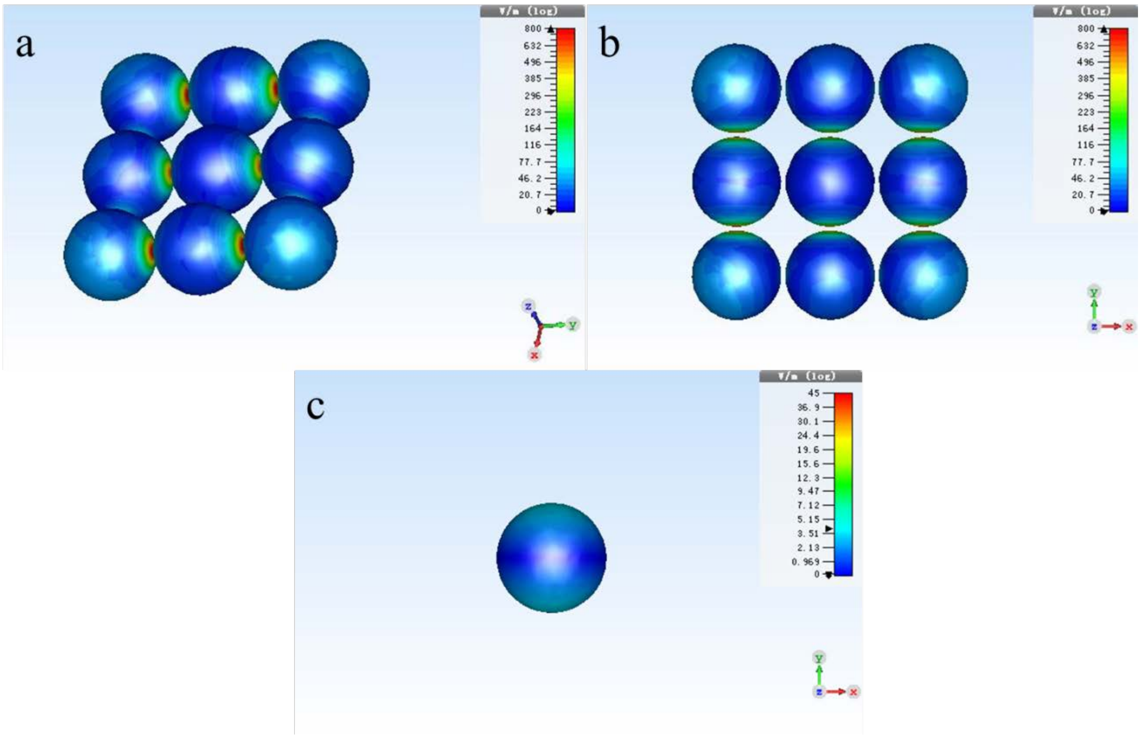

Supplementary Figure15 | Electric field simulation of Au NP-film and Au NP. a, side view of Au-NP film; b, top view of Au-NP film; c, Au NP. The beam for excitation was set in the z-axis direction at wavelength of 800 nm, the electric field was designed in the y-axis direction with initial values of 1 V/ m.

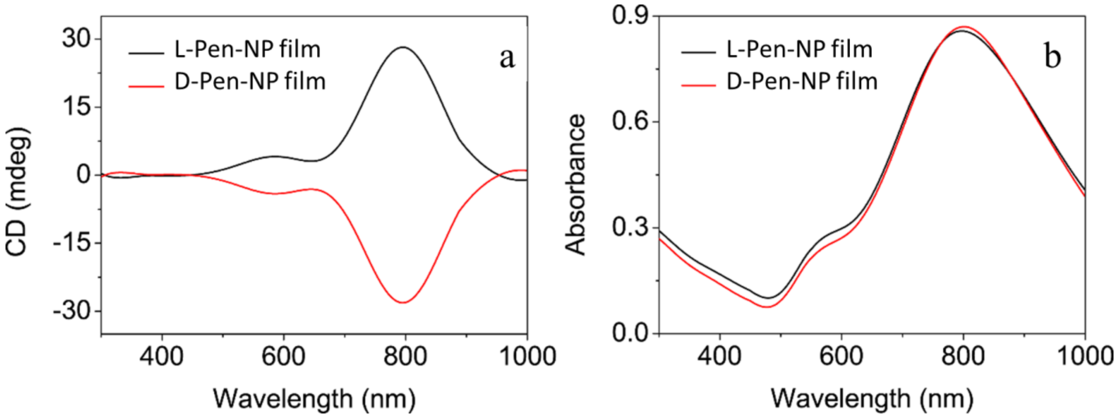

Supplementary Figure16 | Simulated CD (a) and UV-Vis (b) spectra of chiral NP film.

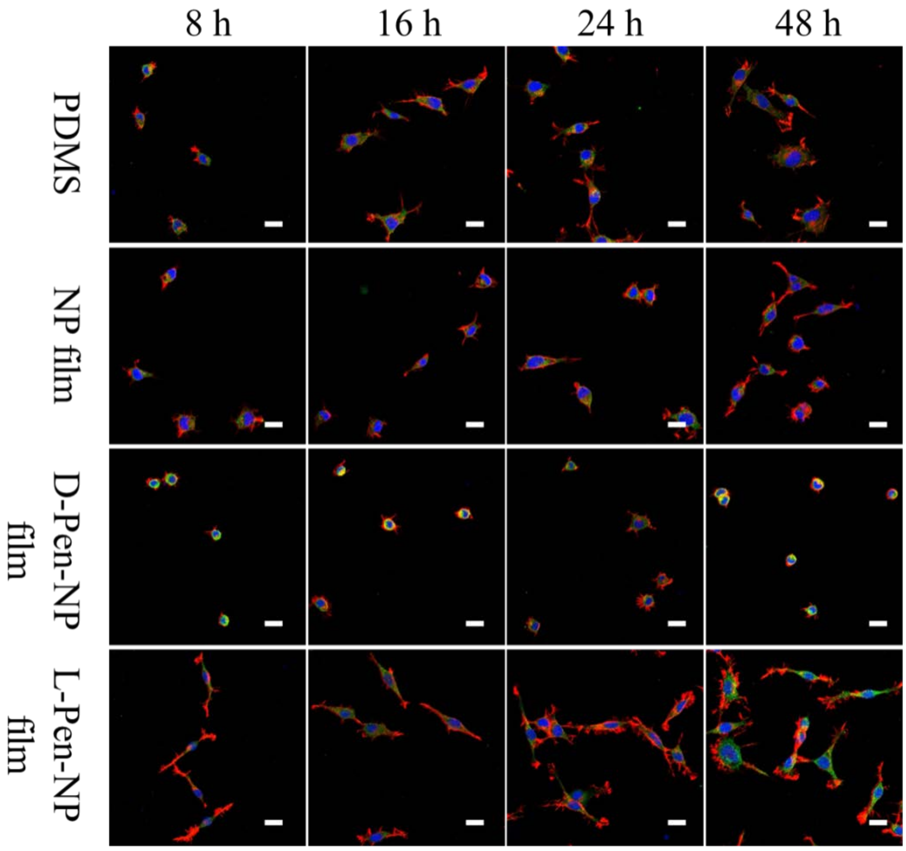

Supplementary Figure 17 | Confocal images of NG108-15 cells cultured on different substrates for different time (red, actin; green, vinculin; blue, nucleus). Scale bar, 20 μm.

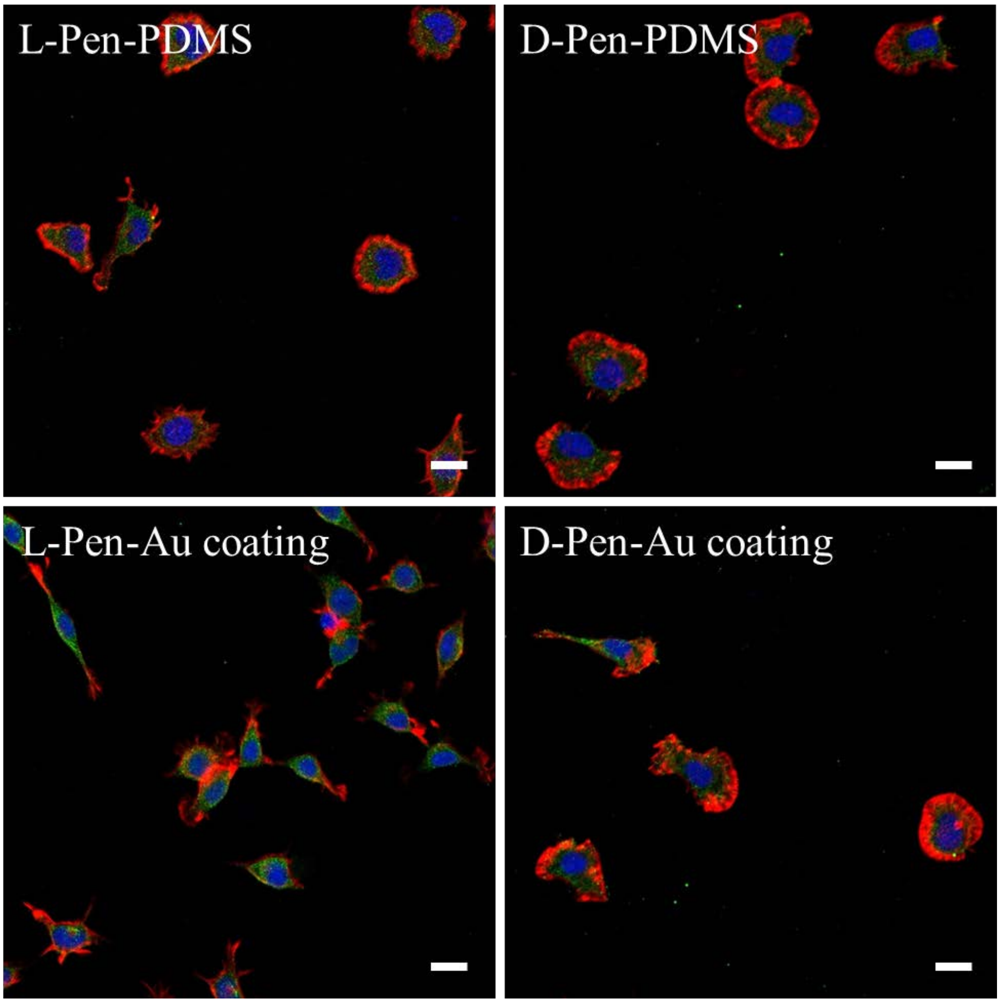

Supplementary Figure18 | Confocal images of NG108-15 cell grown on L- or D-Pen modified PDMS and Au coating (red, actin; green, vinculin; blue, nucleus) for 8 h. Scale bar, 20 μm.

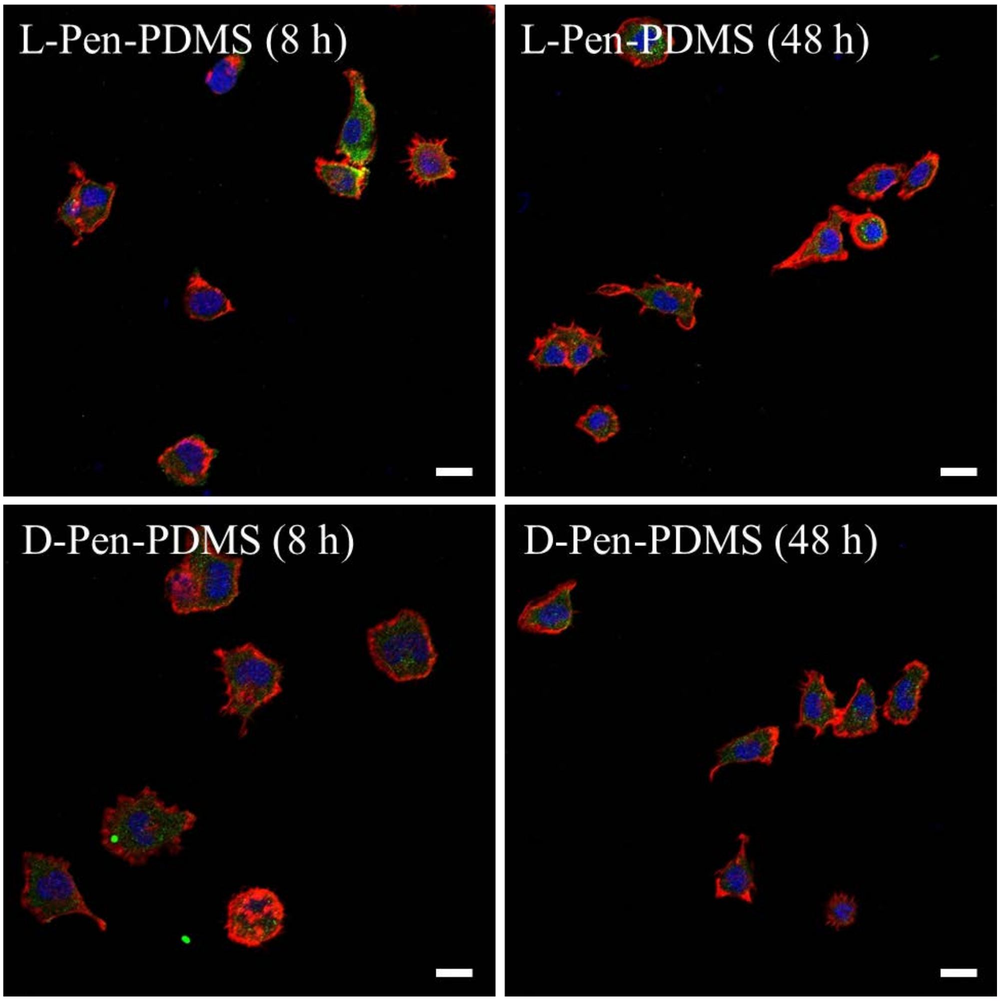

Supplementary Figure19 | Confocal images of NG108-15 cell grown on L or D-Pen modified PDMS.

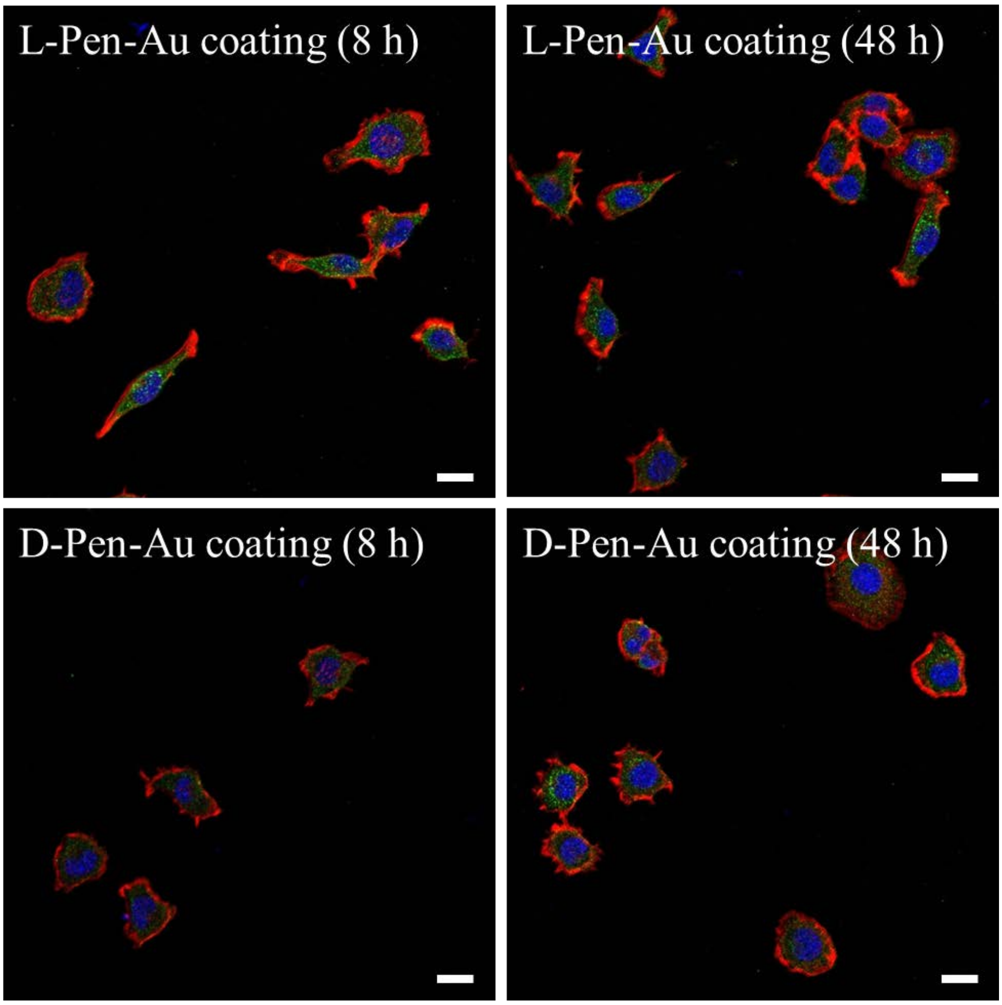

Supplementary Figure 20 | Confocal images of NG108-15 cell grown on L- or D-Pen modified Au coating.

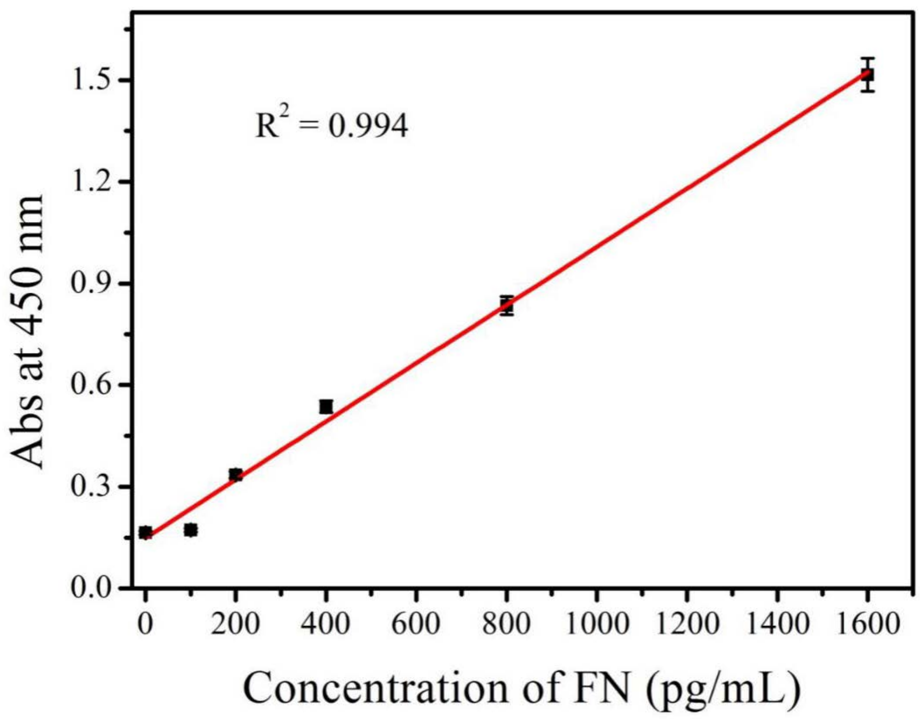

Supplementary Figure21 | A plot of Abs at 450 nm as a function of FN concentrations. The error bars correspond to the standard error of the mean (n=3).

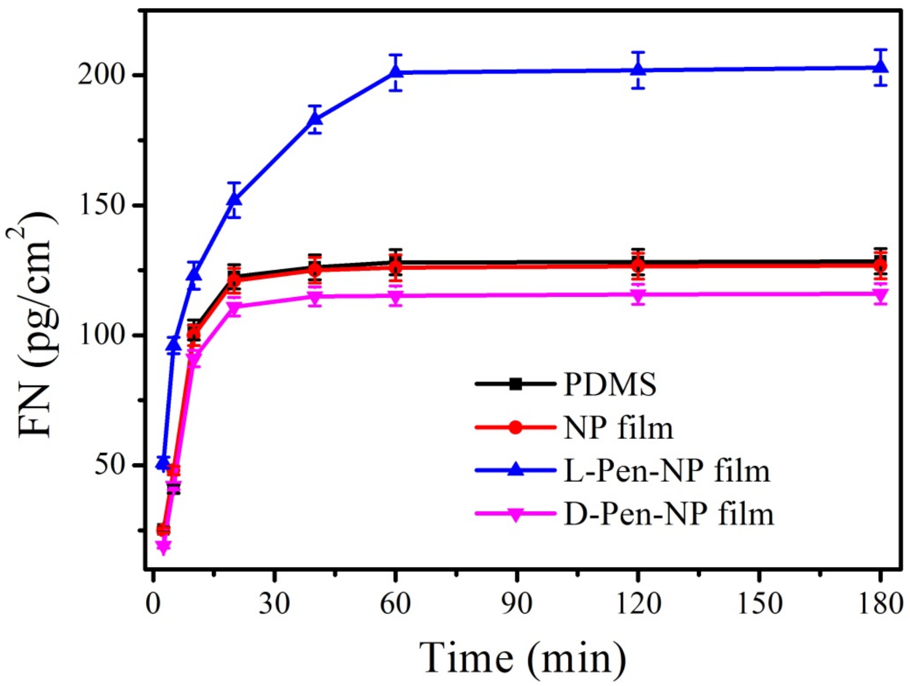

Supplementary Figure22 | Time-dependent adsorption of FN on PDMS, NP film, L-Pen-NP film and D-Pen-NP film. The error bars correspond to the standard error of the mean (n=3).

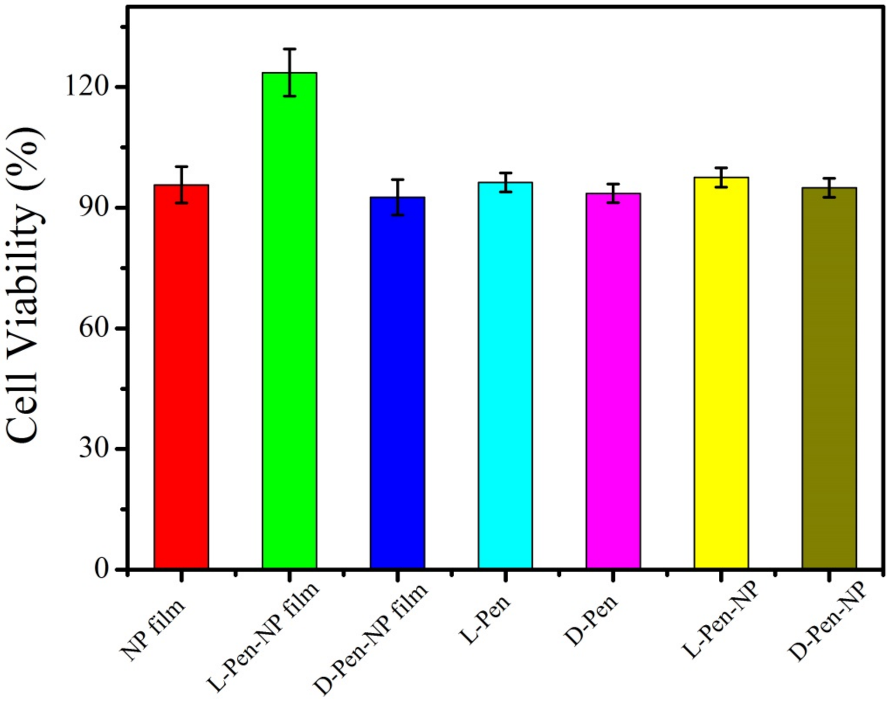

Supplementary Figure 23 | Viability of cell cultured with Au film, Au NP and penicillamine for 48 h. The error bars correspond to the standard error of the mean (n=3).

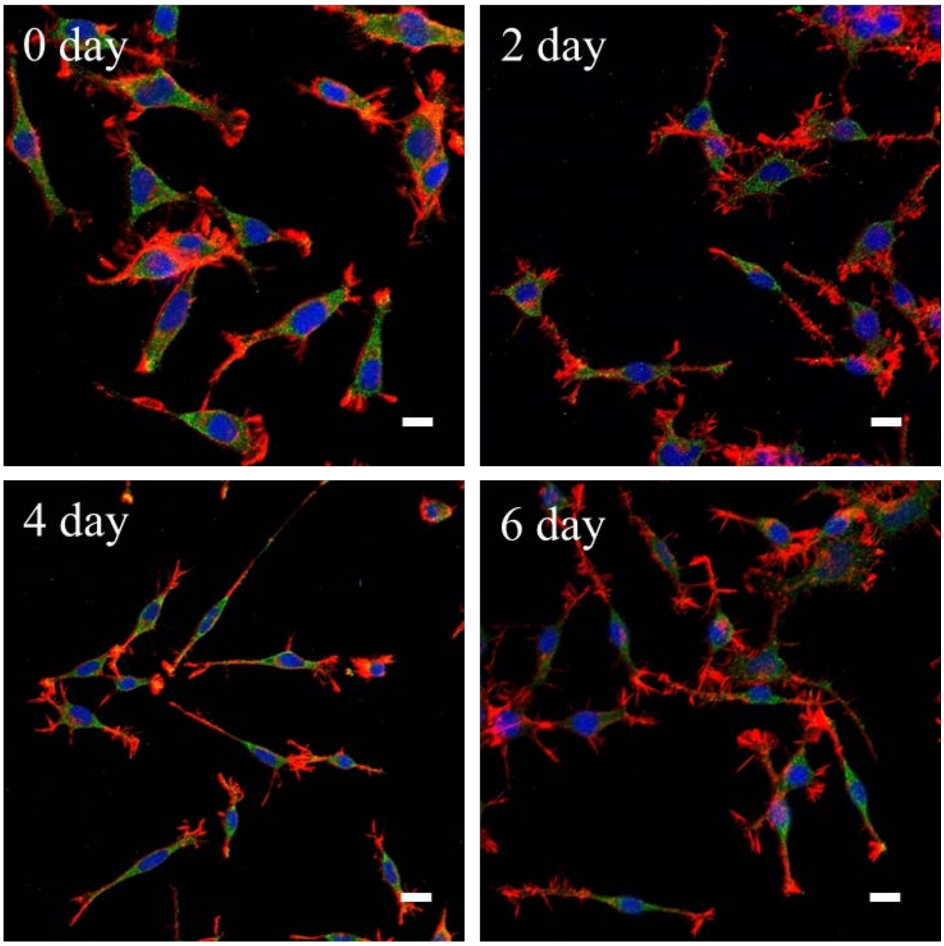

Supplementary Figure 24 | Confocal images of NG108-15 cells cultured on L-Pen-NP film with addition of 1 μM retinoic acid. Scale bar, 20 μm.

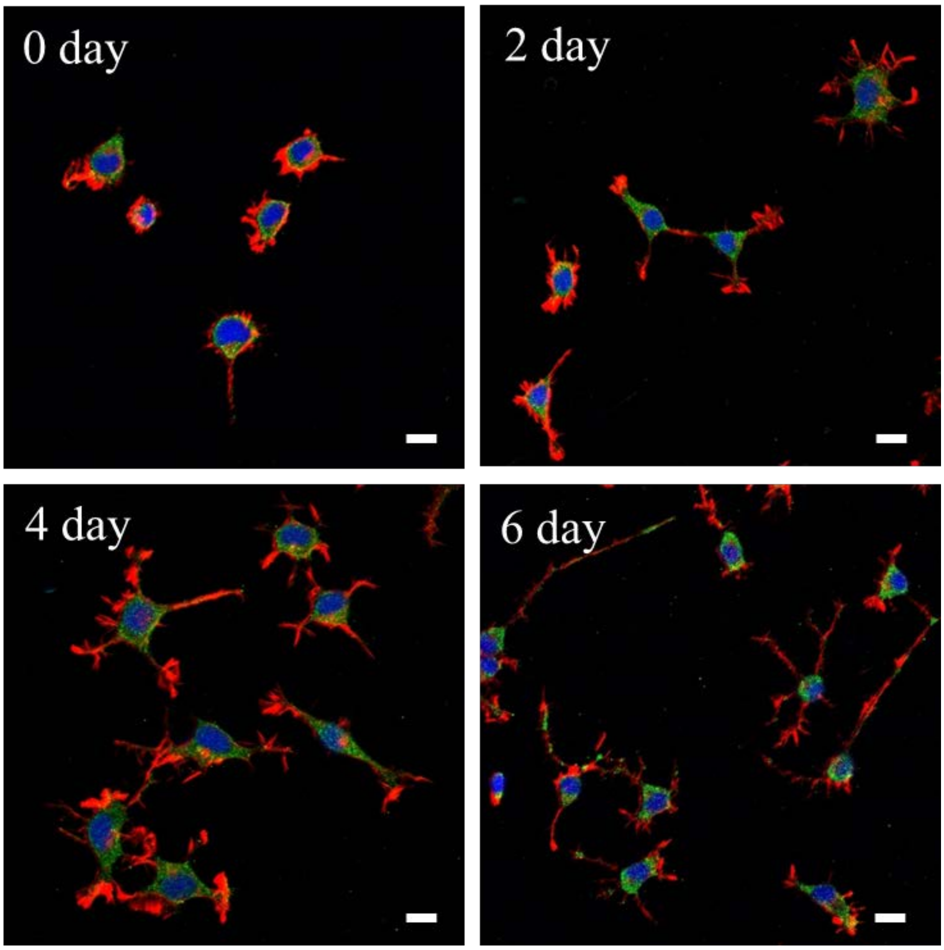

Supplementary Figure 25 | Confocal images of NG108-15 cells cultured on D-Pen-NP film with addition of 1 μM retinoic acid. Scale bar, 20 μm.

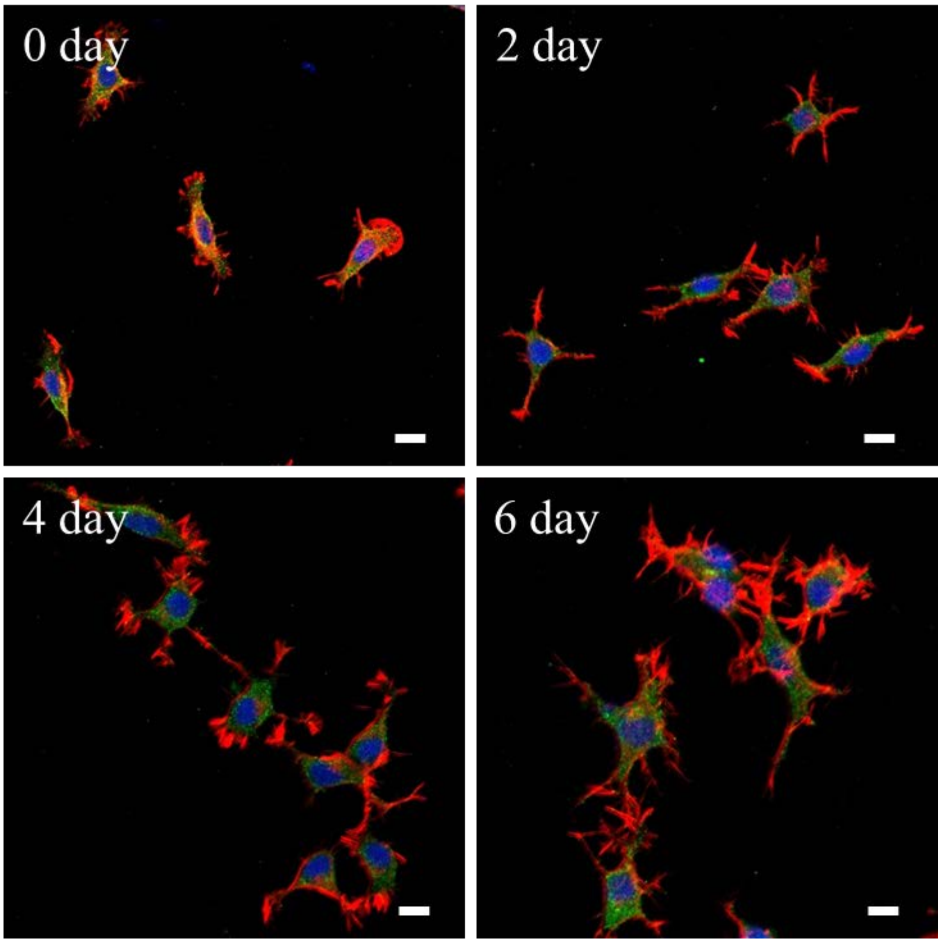

Supplementary Figure 26 | Confocal images of NG108-15 cells cultured on PDMS with addition of 1 μM retinoic acid. Scale bar, 20 μm.

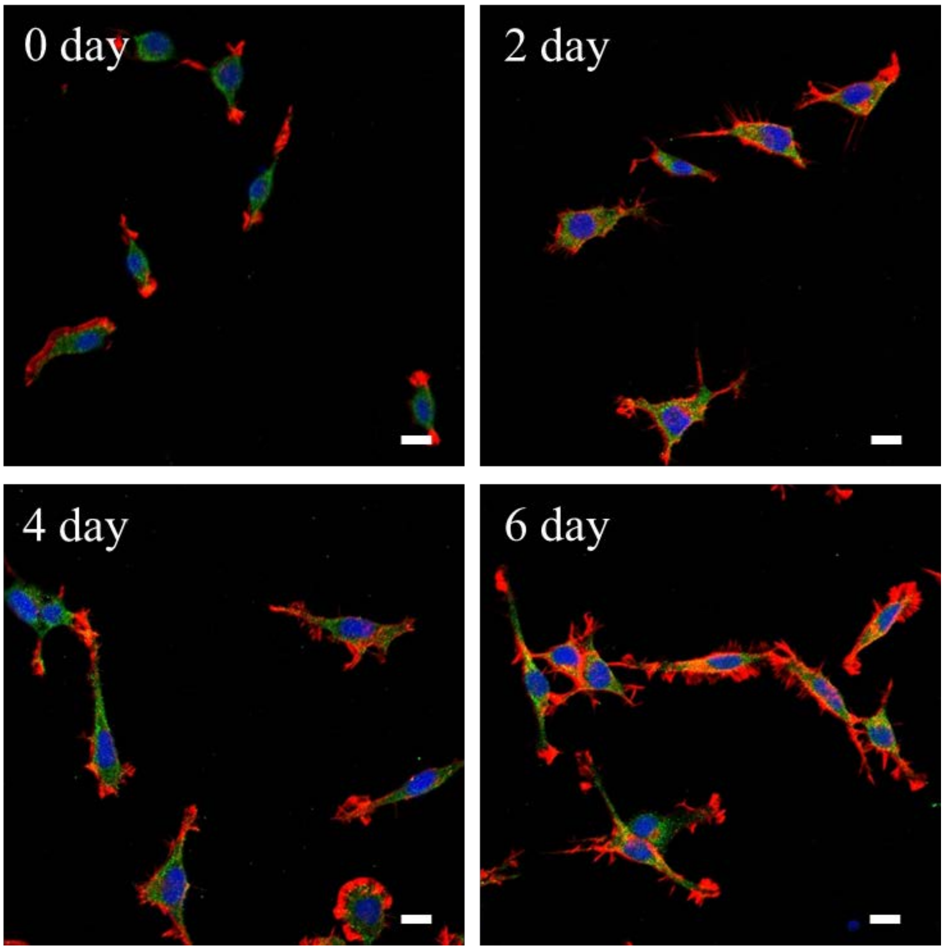

Supplementary Figure 27 | Confocal images of NG108-15 cells cultured on NP-film with addition of 1 μM retinoic acid. Scale bar, 20 μm.

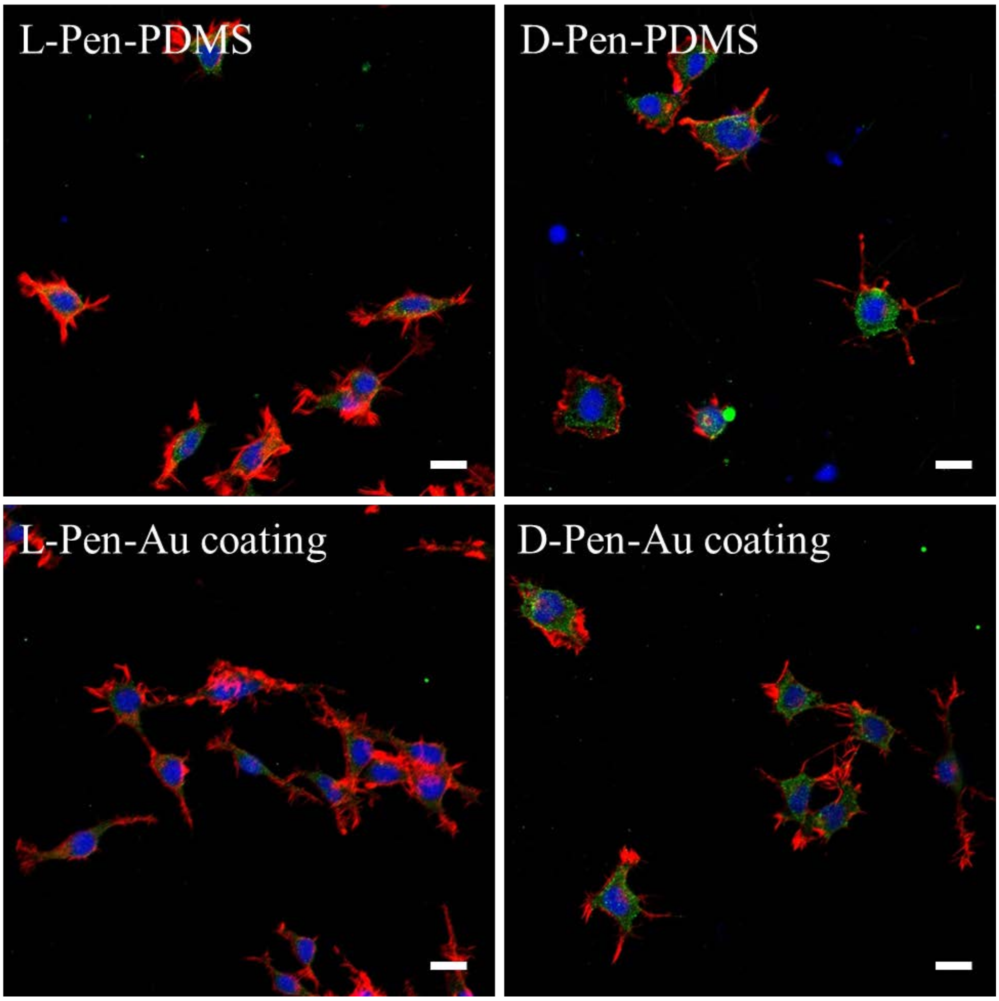

Supplementary Figure 28 | Confocal images of NG108-15 cells with addition of 1 μM retinoic acid. Scale bar, 20 μm.

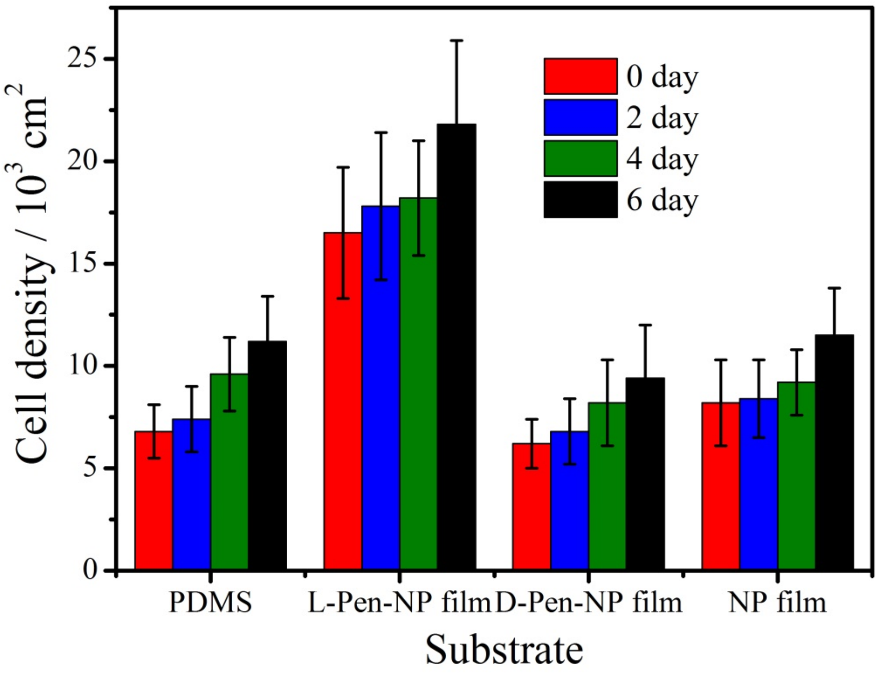

Supplementary Figure 29 | Cell densities of NG108-15 cells cultured on different substrates with addition of retinoic acid for different time. The error bars correspond to the standard error of the mean (n=3).

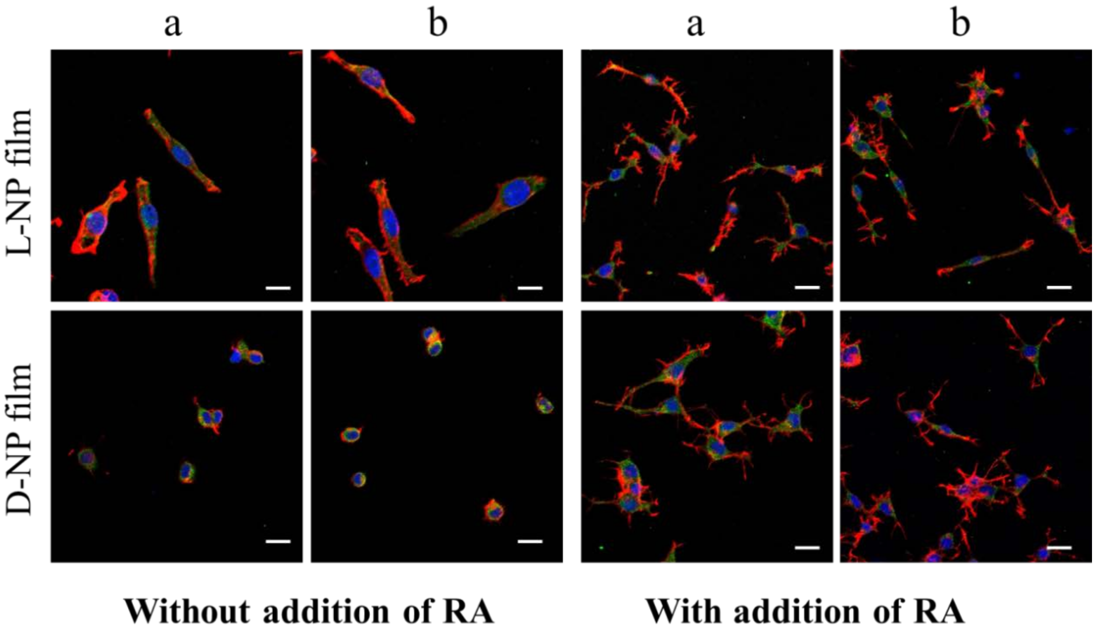

Supplementary Figure 30 | Confocal images of NG108-15 cells adhered on L-NP films and D- NP films functionalized with L/D-cystine (lane a) or L/D-phenylalanine (lane b) without (8 hr) and with addition of retinoic acid (RA, 6 days). (Red, actin; green, vinculin; blue, nucleus.) Scale bars were 20μm.

1. Adherent cells showed stretching when grown on the L-NP film (functionalized with L-cystine or L-phenylalanine), whereas the cells on the D-NP film (functionalized with D-cystine or D-phenylalanine) had a predominantly round morphology.
2. Cells on the L-NP film (functionalized with L-cystine or L-phenylalanine) displayed bipolar differentiation. In contrast, the cells on the D-NP film (functionalized with D-cystine or D-phenylalanine) showed multipolar neurite outgrowth.

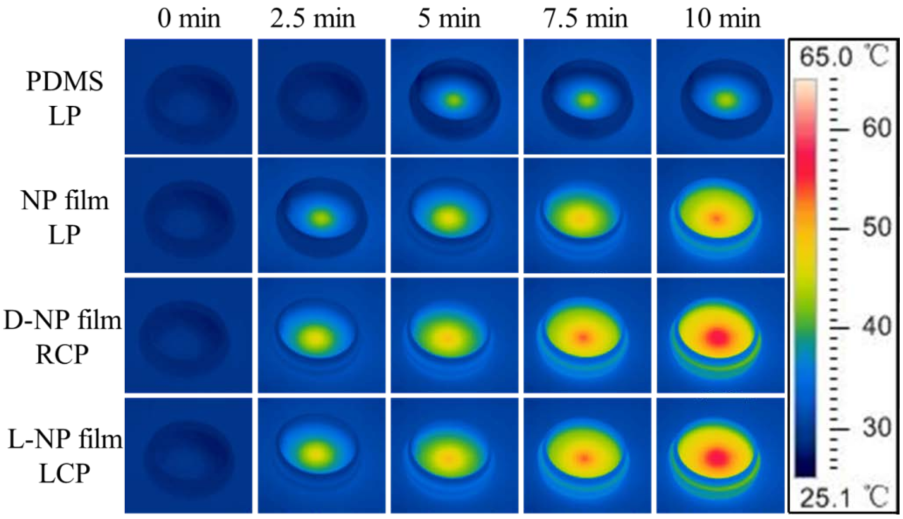

Supplementary Figure 31 | The thermal images of different substrates under the

irradiation of

808 nm.

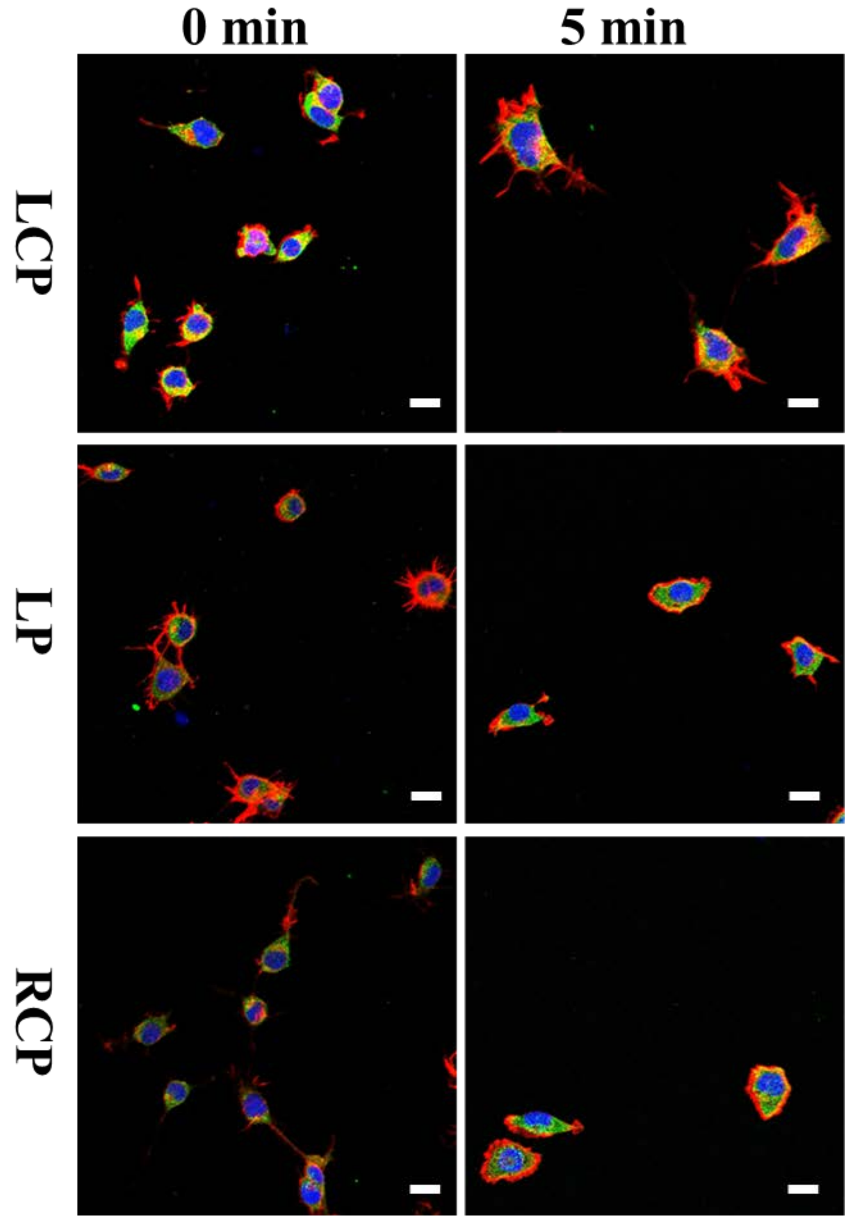

Supplementary Figure 32 | Confocal images of NG108-15 cell grown on NP film upon polarized light irradiation (808nm laser, 150 mW/cm^2). Scale bar, 20 μm.

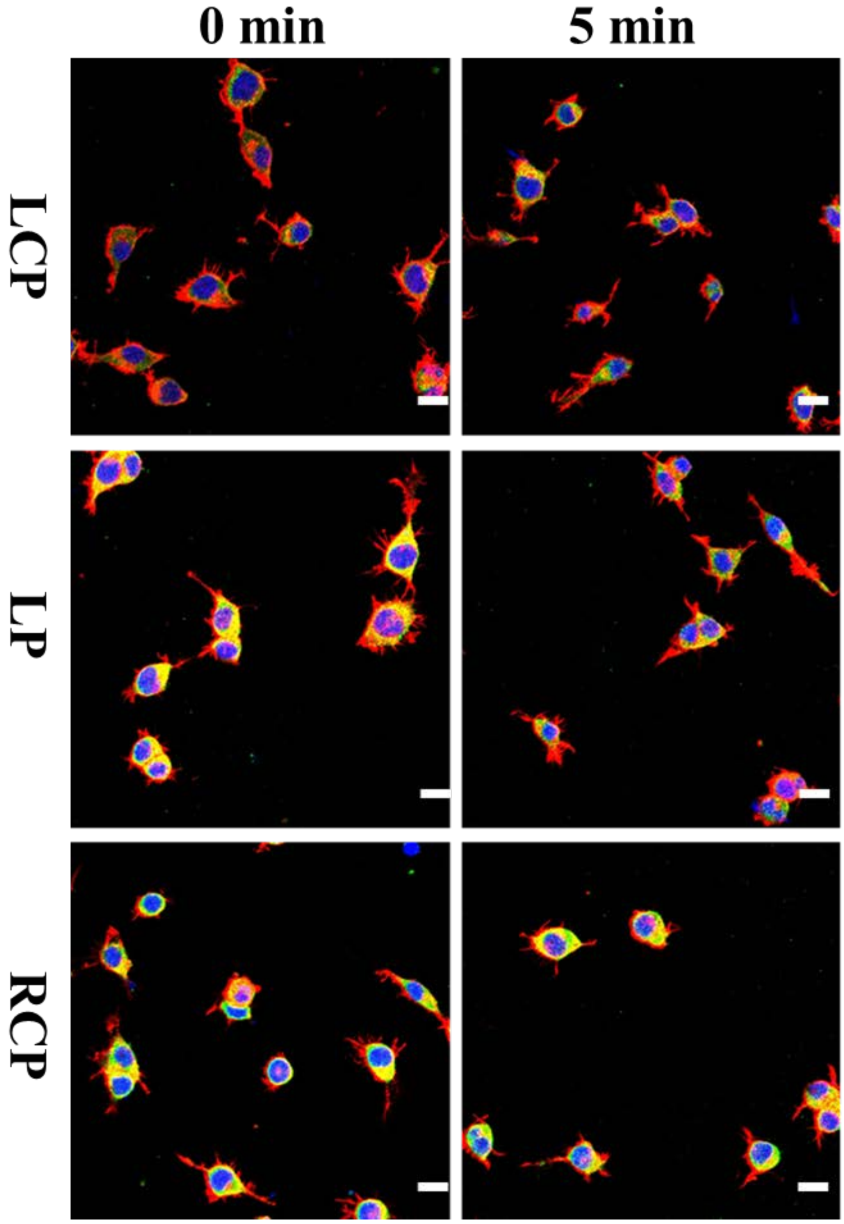

Supplementary Figure 33 | Confocal images of NG108-15 cell grown on PDMS upon polarized light irradiation (808nm laser, 150 mW/cm^2). Scale bar, 20 μm.

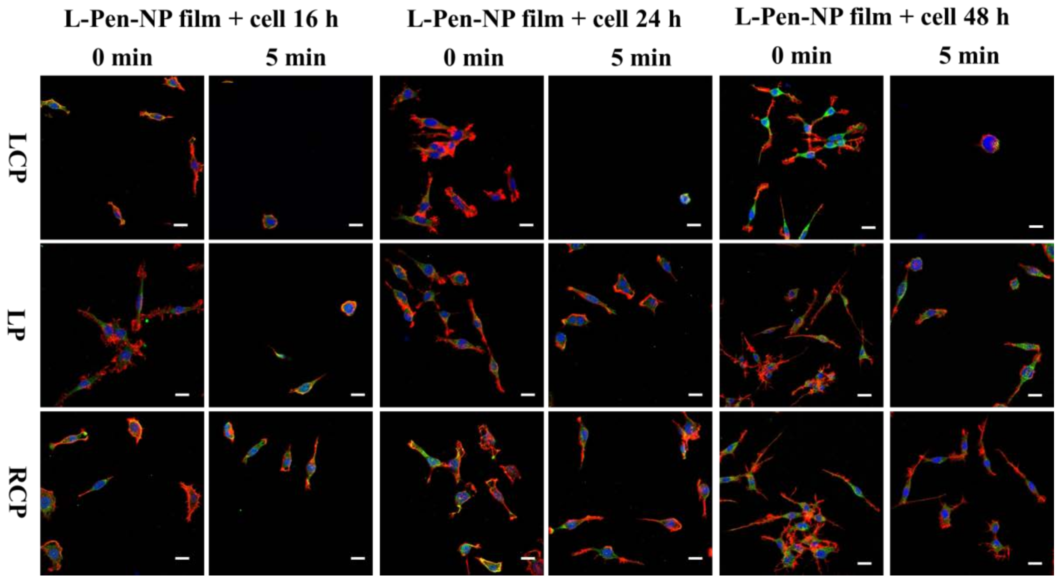

Supplementary Figure 34 | Confocal images of NG108-15 cells grown on L-Pen-NP film for 16, 24 and 48 h, upon polarized light irradiation (808nm laser, 150 mW/cm^2). (Red, actin; green, vinculin; blue, nucleus). Scale bars were 20 μm.

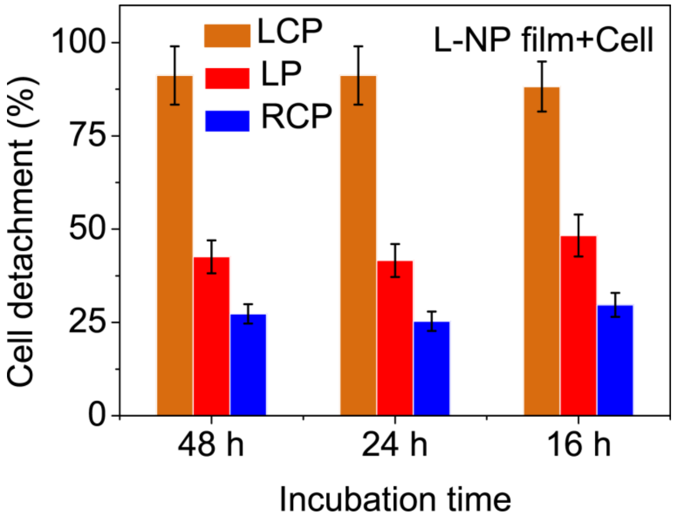

Supplementary Figure 35 | Cell detachment rates after laser irradiation. NG108-15 cells, after cultured on L-Pen-NP film for 48 (as in **Figure 5a**), 24 and 16 h (as in **Supplementary Figure 34**), and then were detached under the illumination of near-infrared light (808nm laser, 150 mW/cm^2, 5 min). The error bars correspond to the standard error of the mean (n=3).

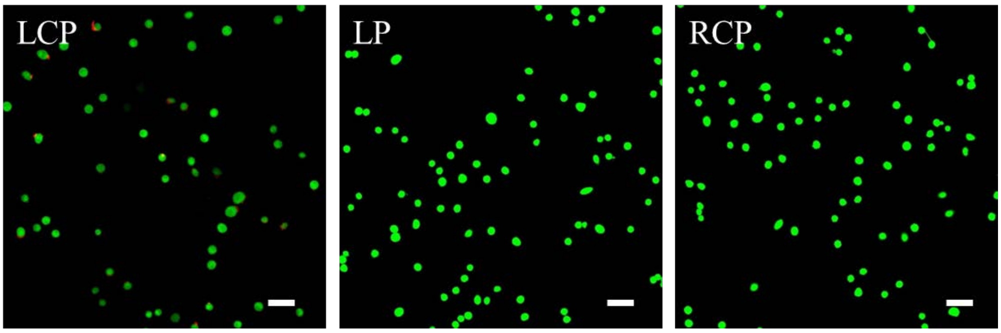

Supplementary Figure 36 | Live/dead assay (green, live; red, dead) conducted on NG108-15 cells which are removed from L-Pen-NP film after 5 min laser excitation (150 mW/cm^2). Scale bar, 50 μm.

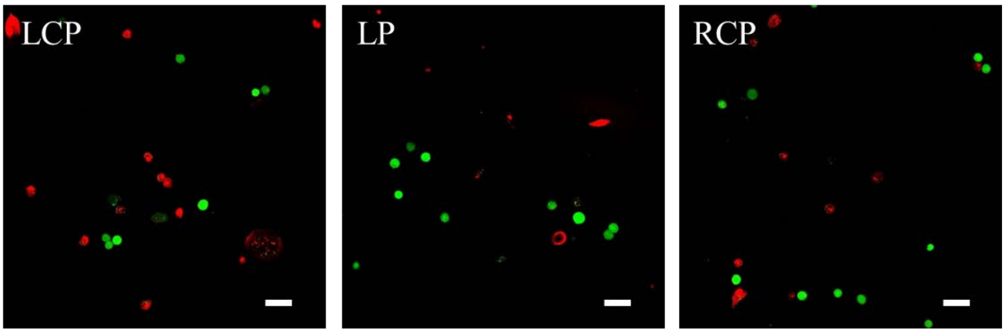

Supplementary Figure 37 | Live/dead assay (green, live; red, dead) conducted on NG108-15 cells which are removed from D-Pen-NP film after 5 min laser excitation (150 mW/cm^2). Scale bar, 50 μm.

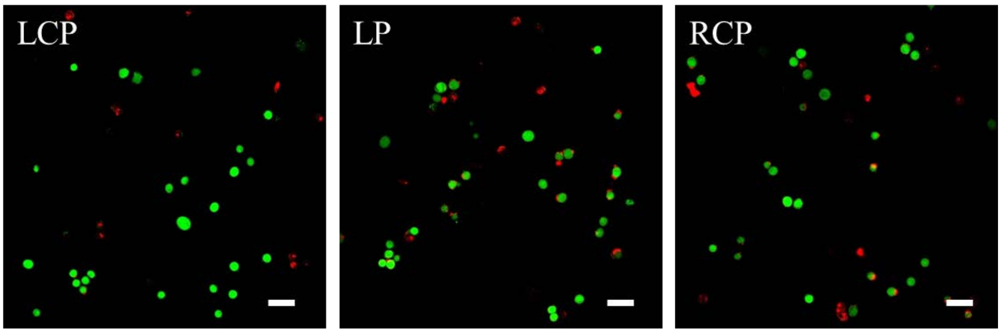

Supplementary Figure 38 | Live/dead assay (green, live; red, dead) conducted on NG108-15 cells which are removed from NP film after 5 min laser excitation (150 mW/cm^2). Scale bar, 50 μm.

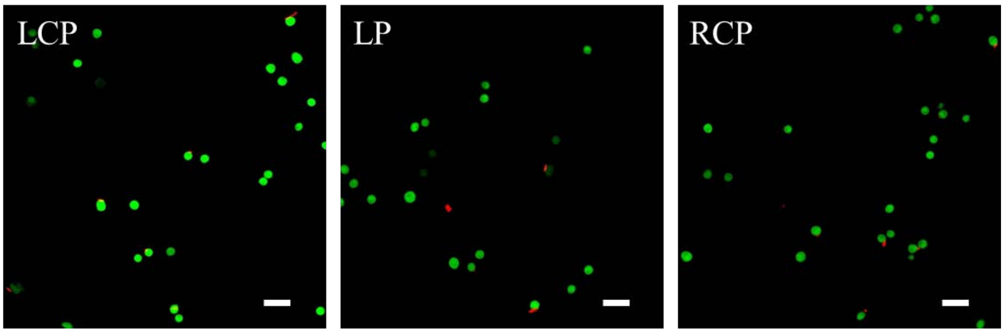

Supplementary Figure 39 | Live/dead assay (green, live; red, dead) conducted on NG108-15 cells which are removed from PDMS after 5 min laser excitation (150 mW/cm^2). Scale bar, 50 μm.

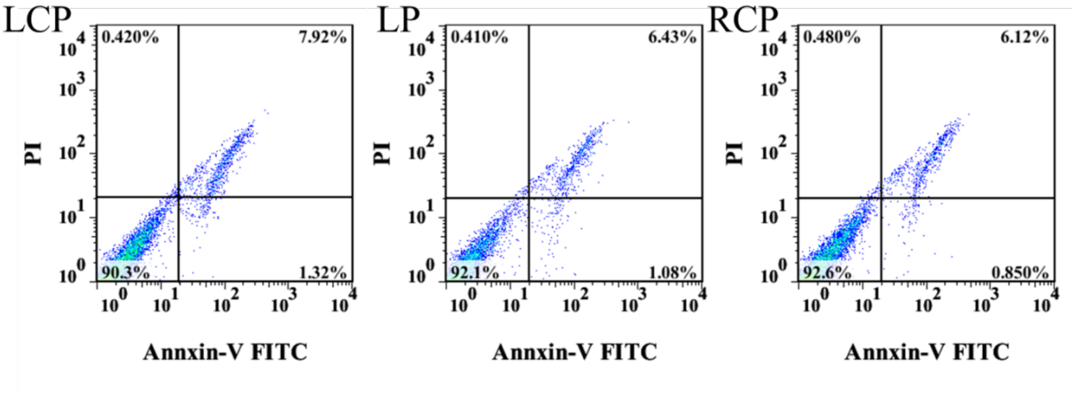

Supplementary Figure 40 | Flow-cytometric analysis of apoptosis in NG108-15 cells removed from L-Pen-NP film surfaces after 5 min laser excitation.

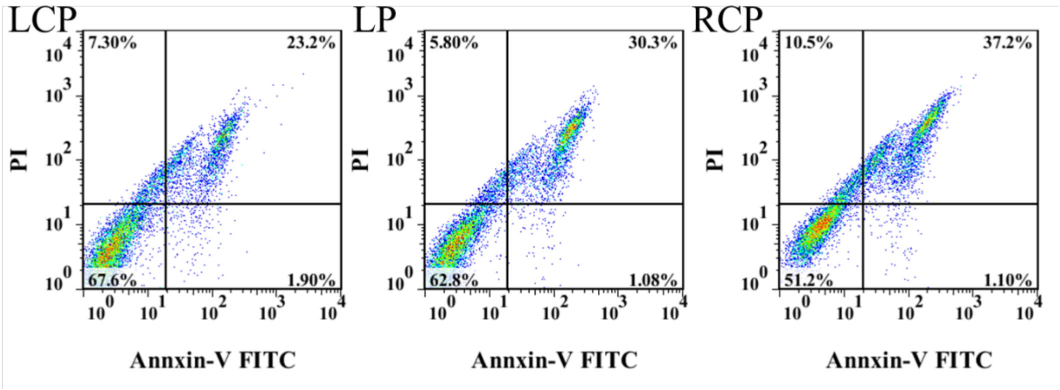

Supplementary Figure 41 | Flow-cytometric analysis of apoptosis in NG108-15 cells removed from D-Pen-NP film surfaces after 5 min laser excitation.

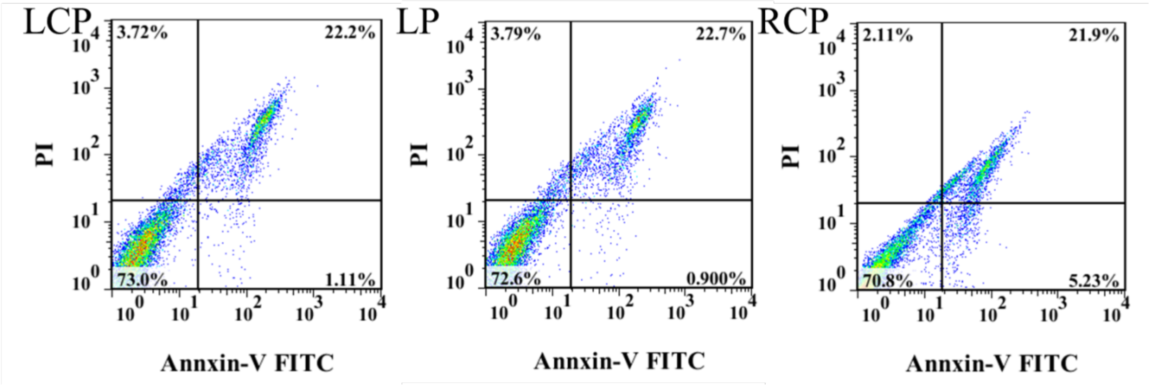

Supplementary Figure 42 | Flow-cytometric analysis of apoptosis in NG108-15 cells removed from NP-film surfaces after 5 min laser excitation.

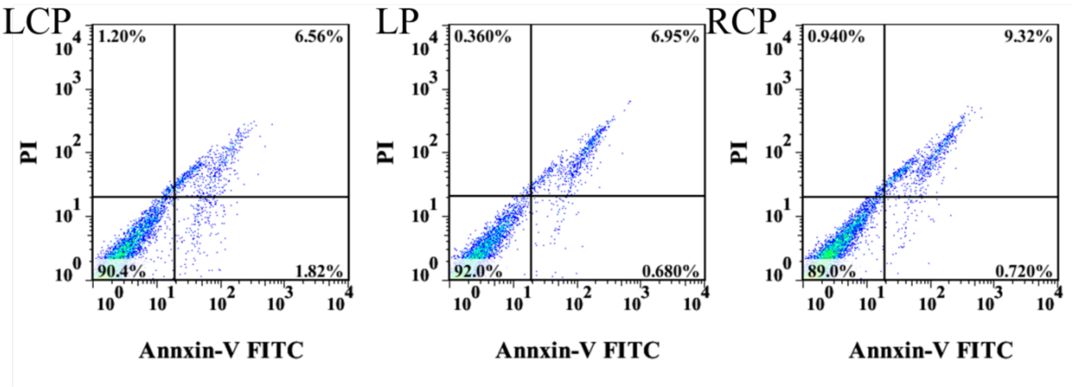

Supplementary Figure 43 | Flow-cytometric analysis of apoptosis in NG108-15 cells removed from PDMS after 5 min laser excitation.

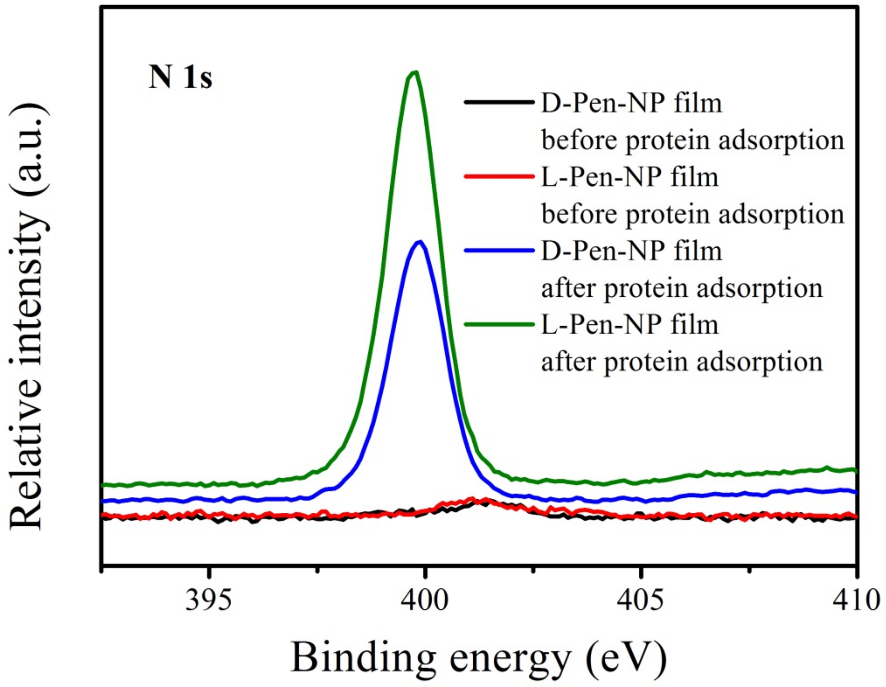

Supplementary Figure 44 | XPS spectra of N element of Au NP films before and after protein (cell medium with 10% fetal bovine serum) adsorption.

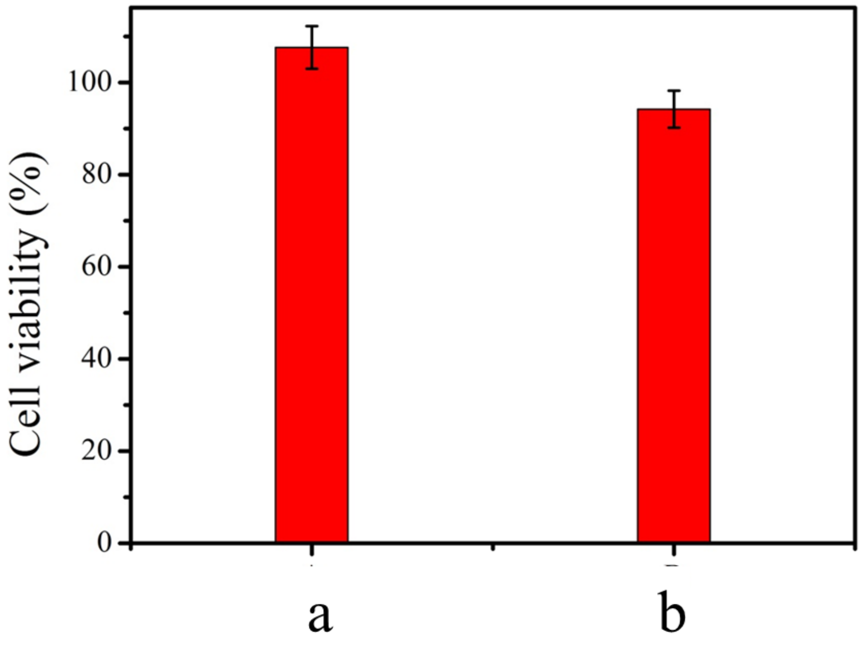

Supplementary Figure 45 | MTT assay of cells detached by NIR light and transferred to another L- (a) and (b) D-Pen-NP film. The error bars correspond to the standard error of the mean (n=3).

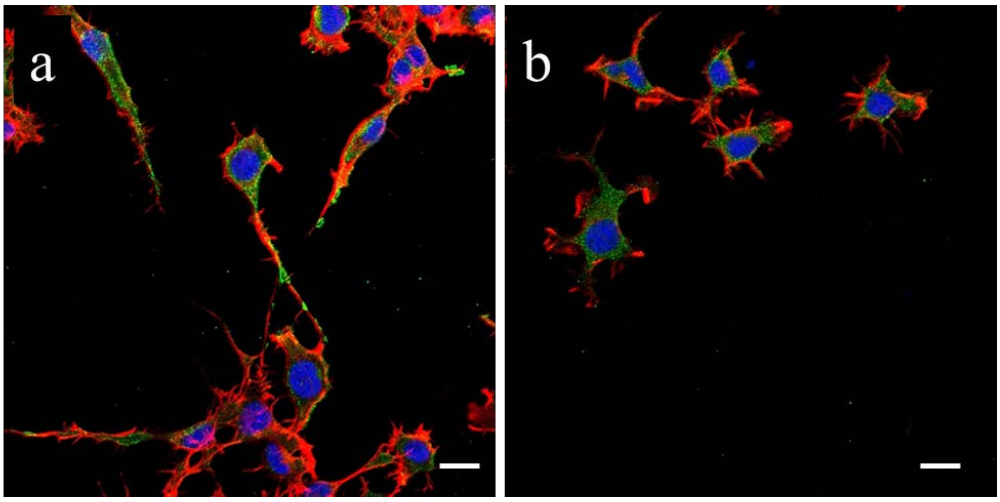

Supplementary Figure 46 | Confocal images of cells detached by NIR light and transferred to another L- (a) and (b) D-Pen-NP film then differentiated (with addition of retinoic acid). Red, actin; green, vinculin; blue, nucleus. Scale bars were 20μm.

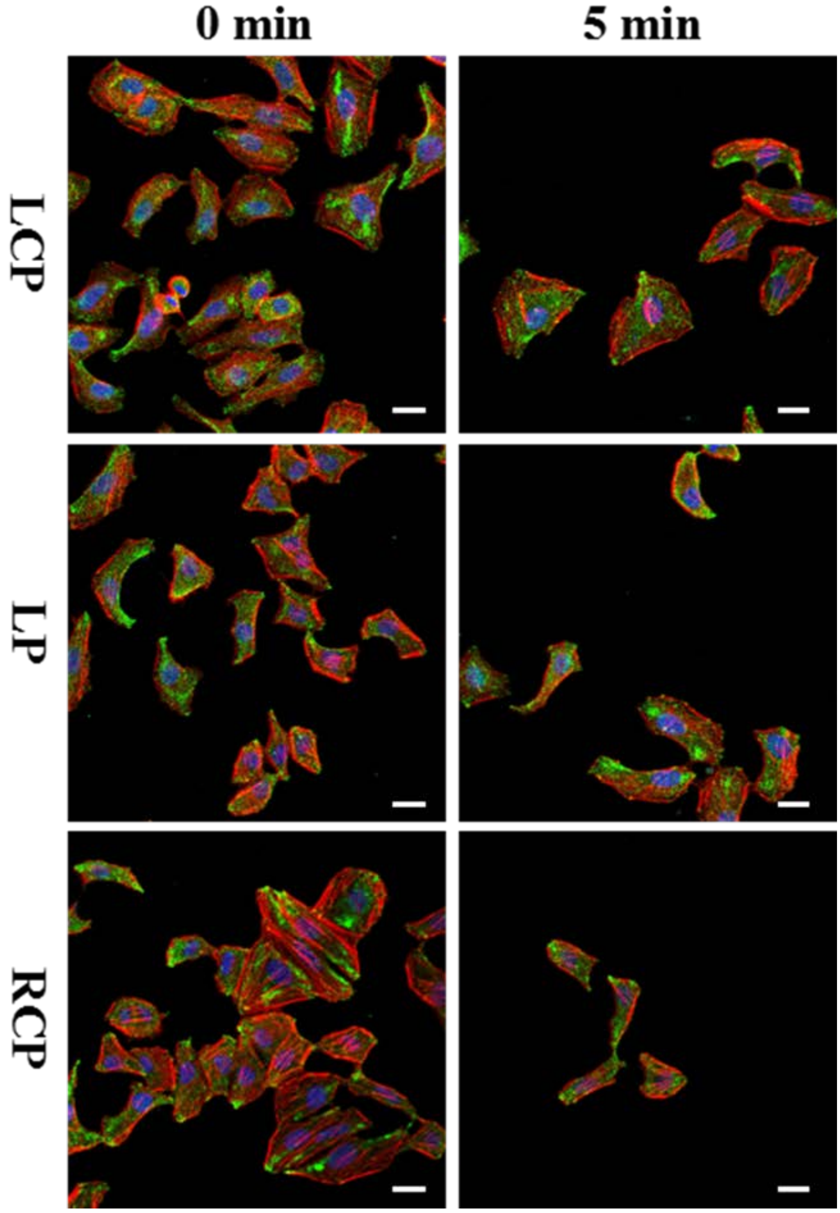

Supplementary Figure 47 | Confocal images of HeLa cell grown on NP film upon polarized light irradiation (808nm laser, 150 mW/cm^2). Scale bar, 25 μm.

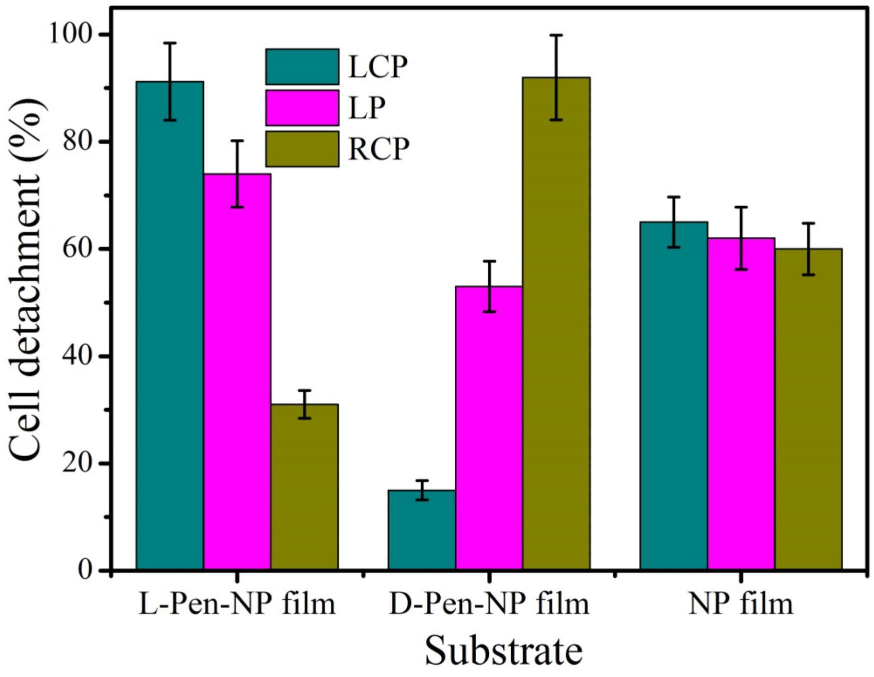

Supplementary Figure 48 | HeLa cell detachment rates upon 808 nm laser irradiation.

The error bars correspond to the standard error of the mean (n=3).

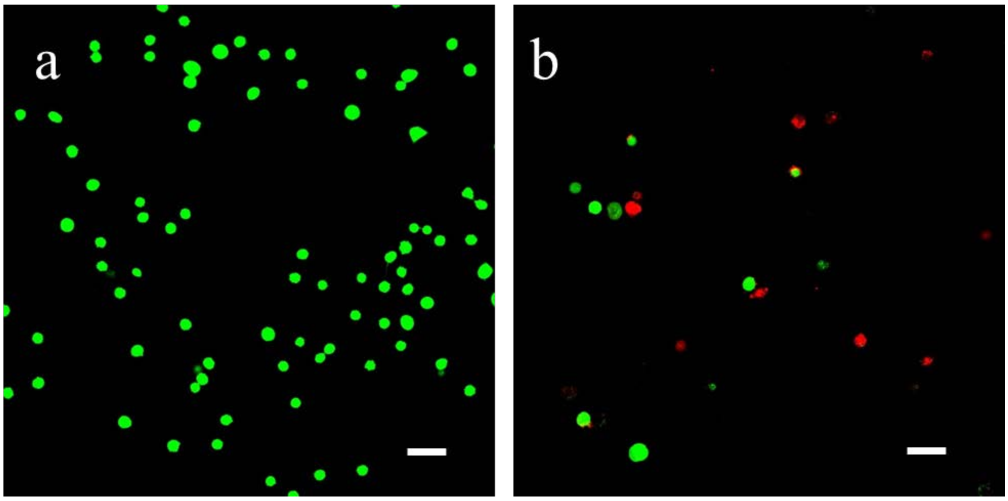

Supplementary Figure 49 | Live/dead assay (green, live; red, dead) conducted on HeLa cells which are removed from a) L-Pen-NP film surfaces under LCP irradiation and b) D-Pen-NP film surfaces under RCP irradiation. Scale bar, 50 μm.

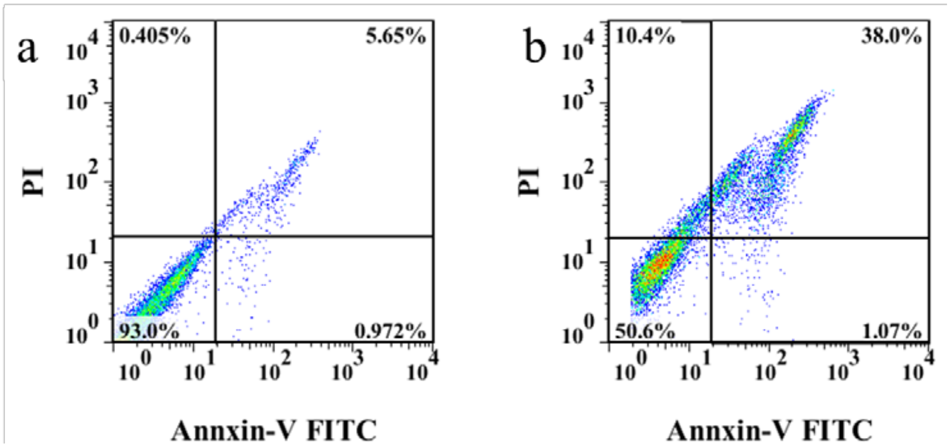

Supplementary Figure 50 | Flow-cytometric analysis of apoptosis in HeLa cells removed from a) L-Pen-NP film surfaces under LCP irradiation and b) D-Pen-NP film surfaces under RCP irradiation.

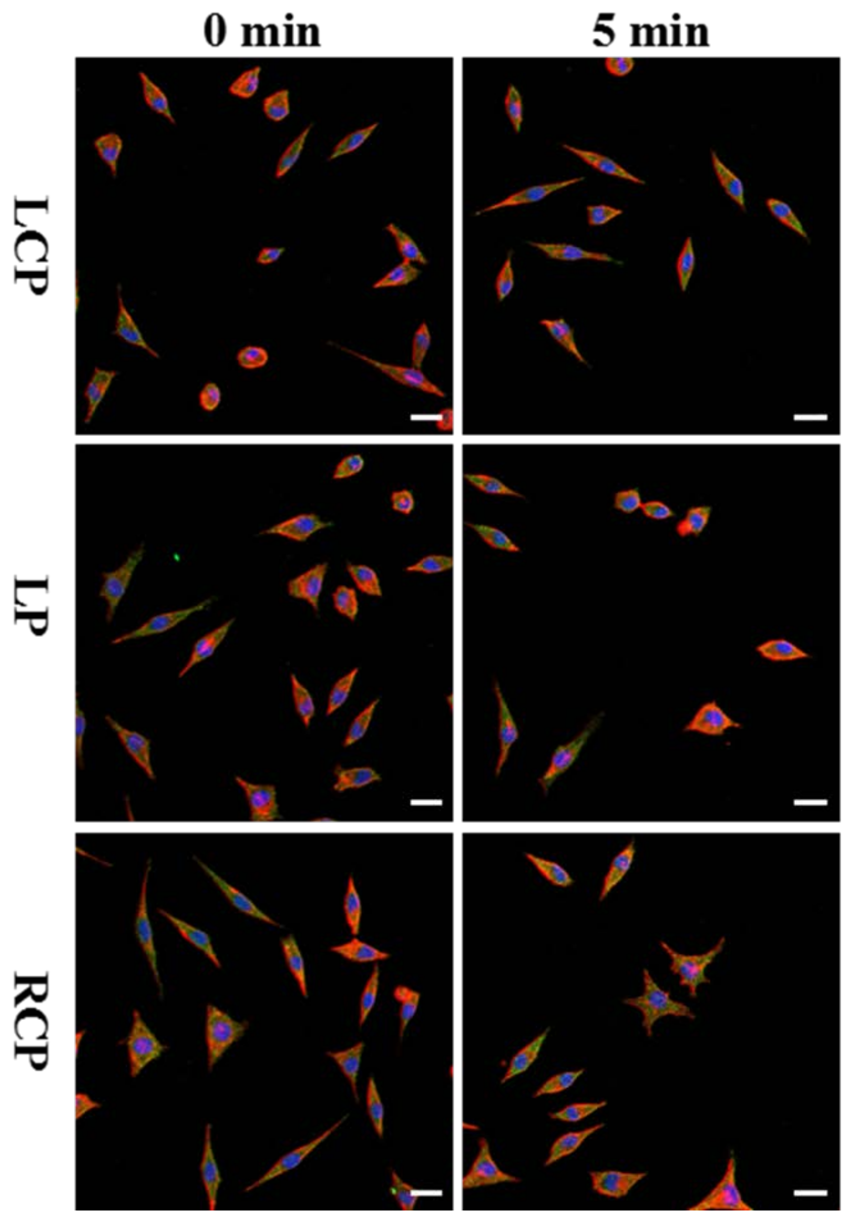

Supplementary Figure 51 | Confocal images of PCS-460-010 cell grown on NP film upon polarized light irradiation (808nm laser, 150 mW/cm^2). Scale bar, 25 μm.

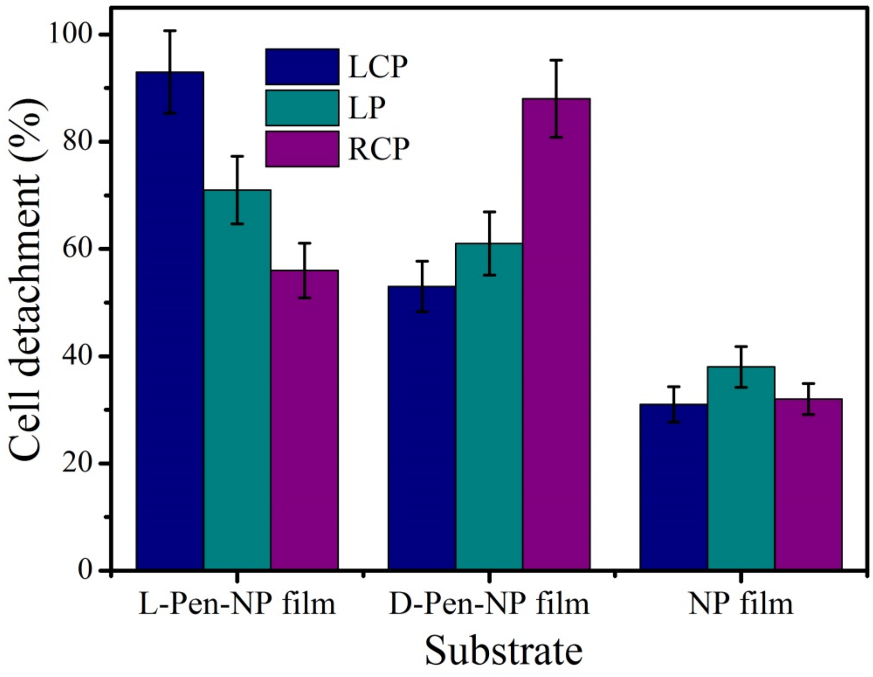

Supplementary Figure 52 | PCS-460-010 Cell detachment rates upon 808 nm laser irradiation. The error bars correspond to the standard error of the mean (n=3).

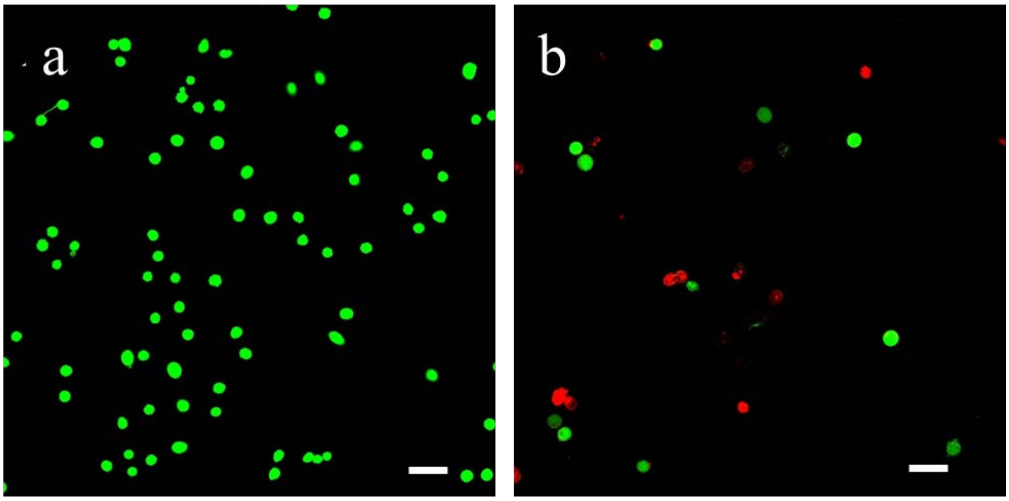

Supplementary Figure 53 | Live/dead assay (green, live; red, dead) conducted on PCS-460-010 cells which are removed from a) L-Pen-NP film surfaces under LCP irradiation and b) D-Pen-NP film surfaces under RCP irradiation. Scale bar, 50 μm.

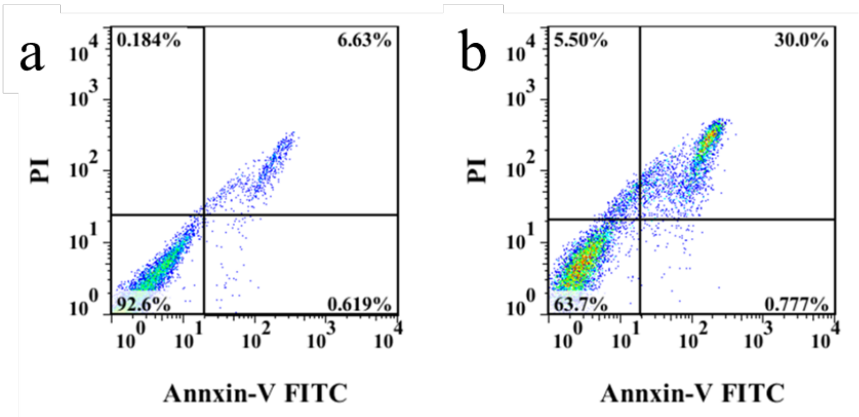

Supplementary Figure 54 | Flow-cytometric analysis of apoptosis in PCS-460-010 cells removed from a) L-Pen-NP film surfaces under LCP irradiation and b) D-Pen-NP film surfaces under RCP irradiation.

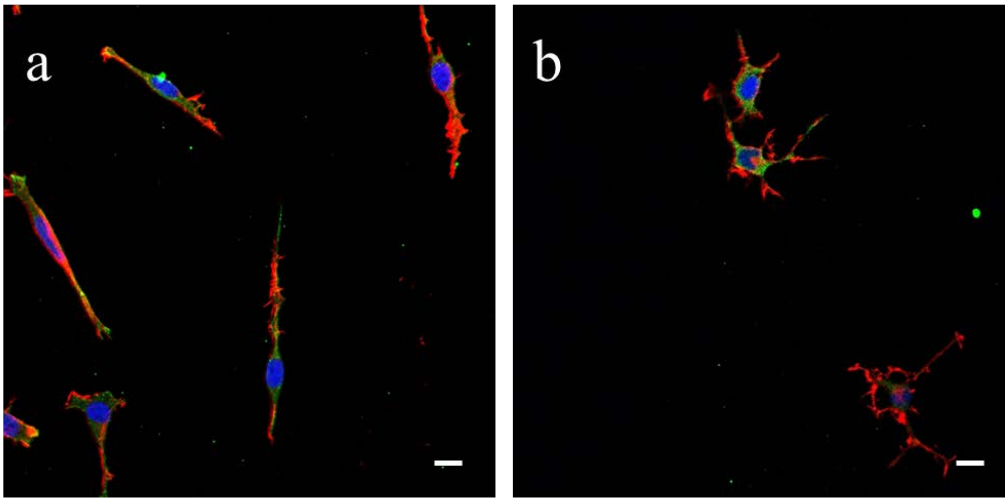

Supplementary Figure 55 | Confocal images of the differentiated cells transferred to normal tissue culture plate 24 h later (a, cells differentiated on L-Pen-NP film and then were transferred to normal tissue culture plate. b, cells differentiated on D-Pen-NP film and then were transferred to normal tissue culture plate.) (red, actin; green, vinculin; blue, nucleus). Scale bars were 20μm.

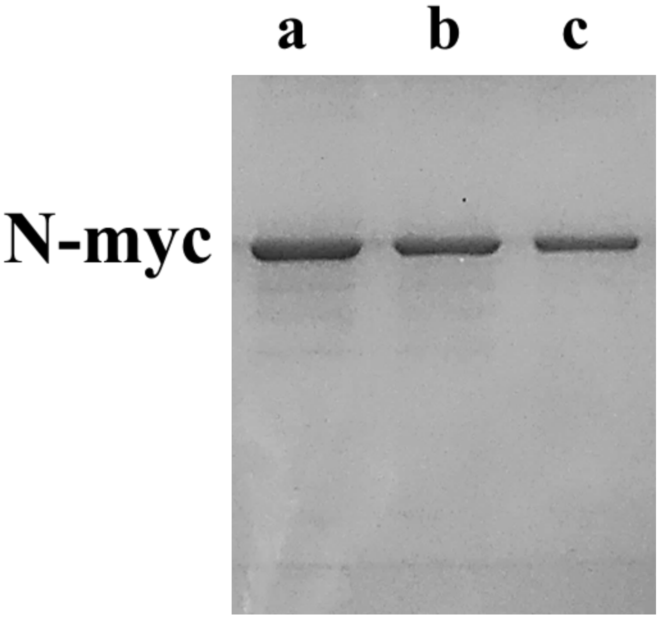

Supplementary Figure 56 | Expression of N-myc protein in the differentiated cells and then transferred to normal tissue culture plate 24 h later (a, control. b, cells differentiated on D-Pen-NP film and then were transferred to normal tissue culture plate. c, cells differentiated on L-Pen-NP film and then were transferred to normal tissue culture plate).

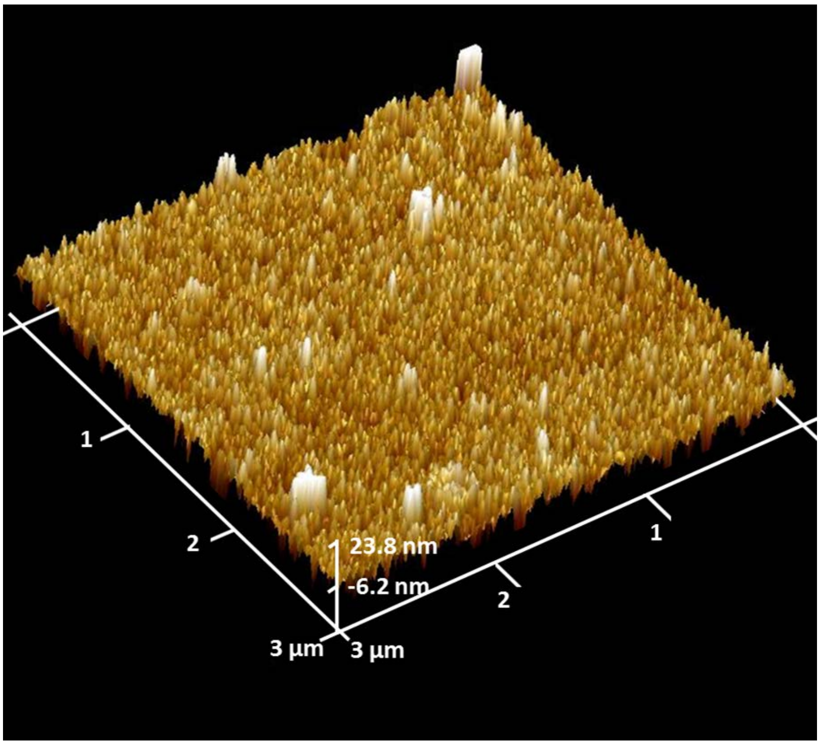

Supplementary Figure 57 | AFM 3D image of Au NP film on glass substrate.

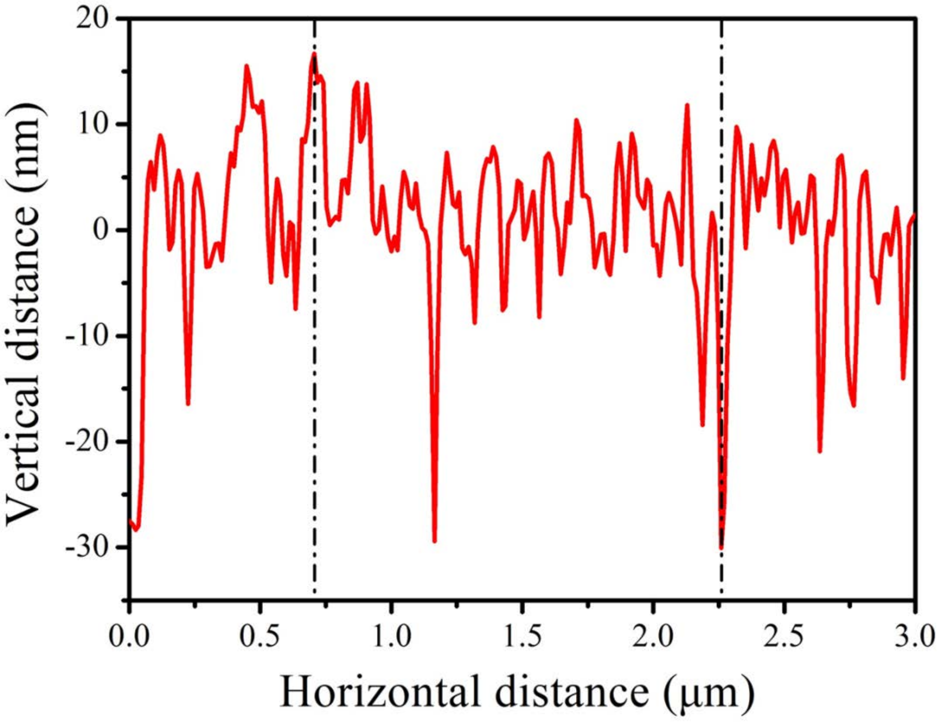

Supplementary Figure 58 | Vertical distance of Au NP film on glass substrate.

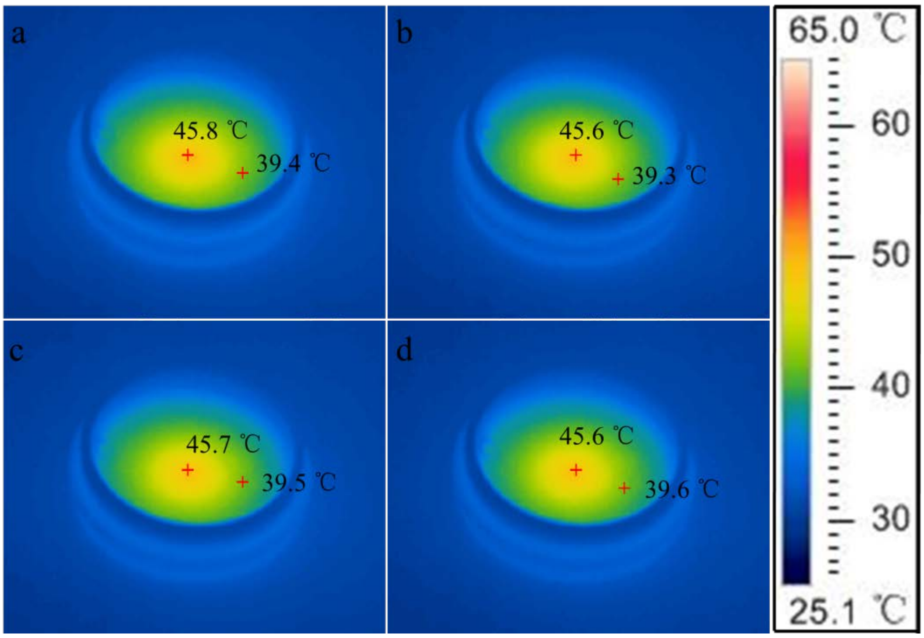

Supplementary Figure 59 | The thermal images of L-Pen-NP film under the irradiation of LCP for 5 min (808nm laser, 150 mW/cm^2).

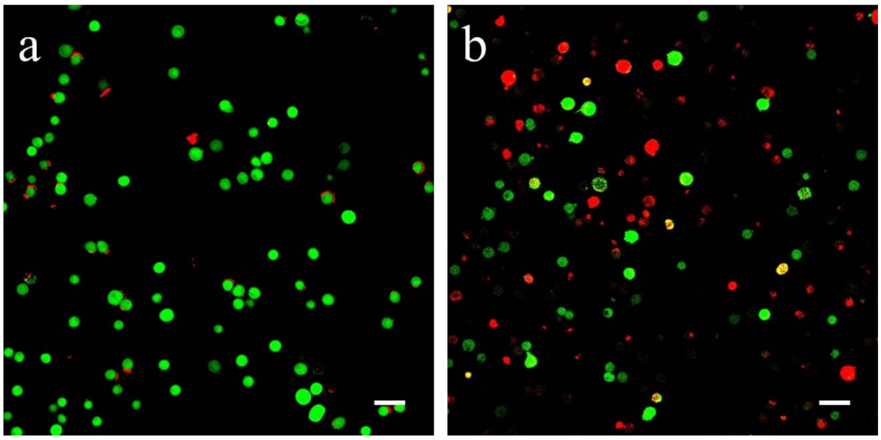

Supplementary Figure 60 | Live/dead assay (green, live; red, dead) conducted on NG108-15 cells which were dropped on the two treated films for LCP illumination (808nm laser, 150 mW/cm^2, 5 min) respectively. a) L-Pen-NP film incubated in cell medium (10% fetal bovine serum, FBS) at 37 °C for 12 h, and b) L-Pen-NP film was incubated in PBS at 37 °C for 12 h as control. Scale bars were 50 μm.

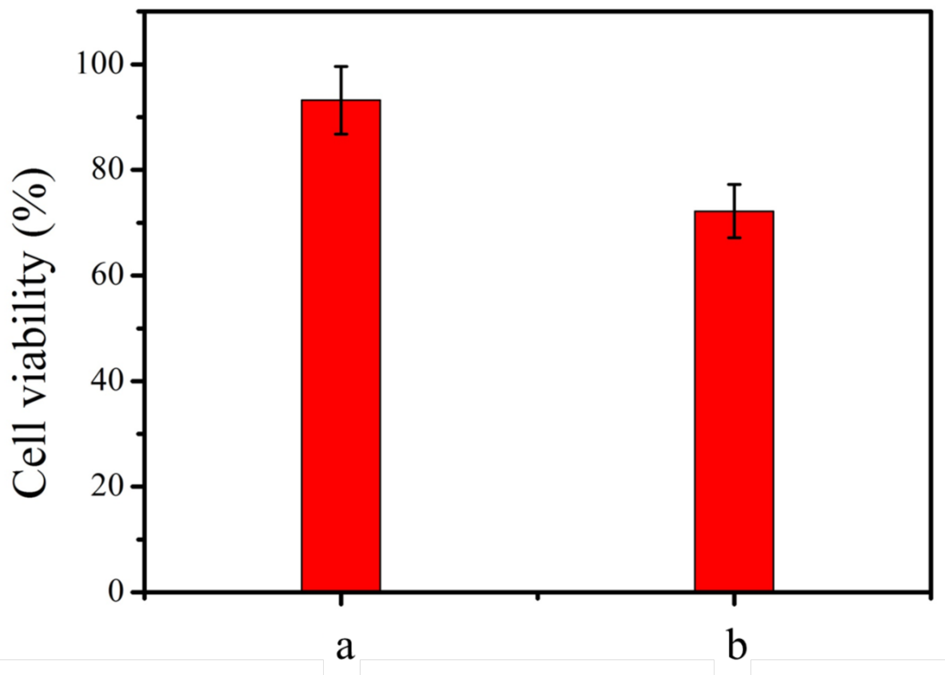

Supplementary Figure 61 | MTT assay conducted on NG108-15 cells which were dropped on the two treated films for LCP illumination (808nm laser, 150 mW/cm^2, 5 min) respectively. a) L-Pen-NP film incubated in cell medium (10% fetal bovine serum, FBS) at 37 °C for 12 h, and b) L-Pen-NP film was incubated in PBS at 37 °C for 12 h as control. The error bars correspond to the standard error of the mean (n=3).

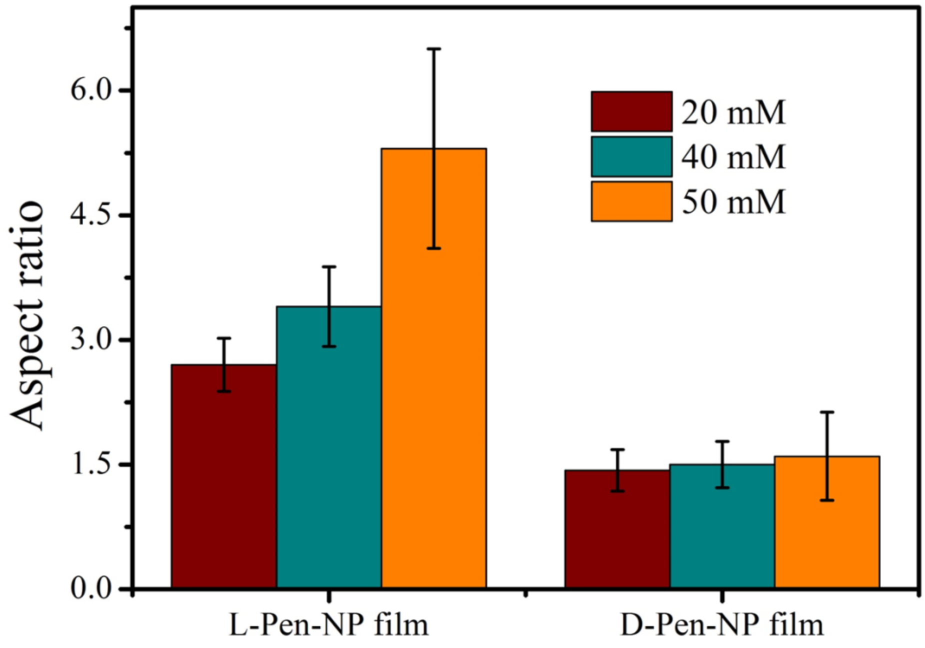

Supplementary Figure 62 | Aspect ratio of NG 108-15 cells cultured on L- or D-Pen-NP film modified with different amounts of L/D-Pen without addition of RA. The error bars correspond to the standard error of the mean (n=6).

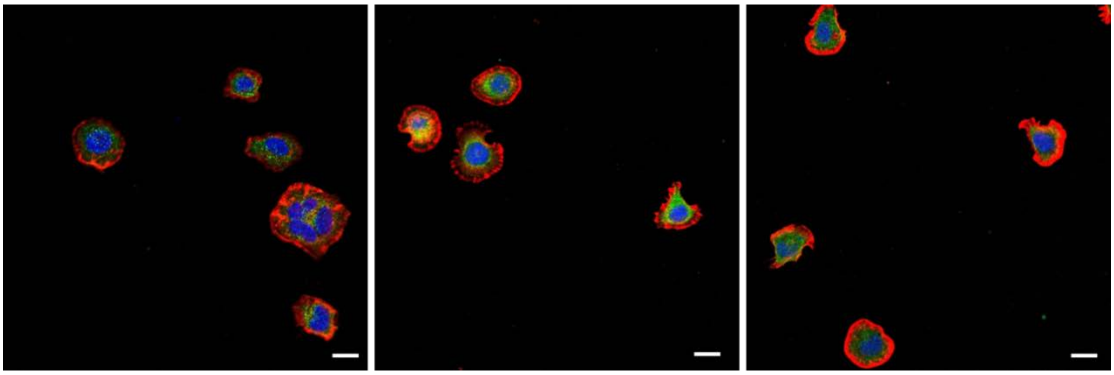

Supplementary Figure 63 | Confocal images of NG108-15 cells adhered on D-Pen-NP films functionalized with 20 mM D-Pen (8 hr) without addition of retinoic acid. (Red, actin; green, vinculin; blue, nucleus). Scale bars were 20μm.

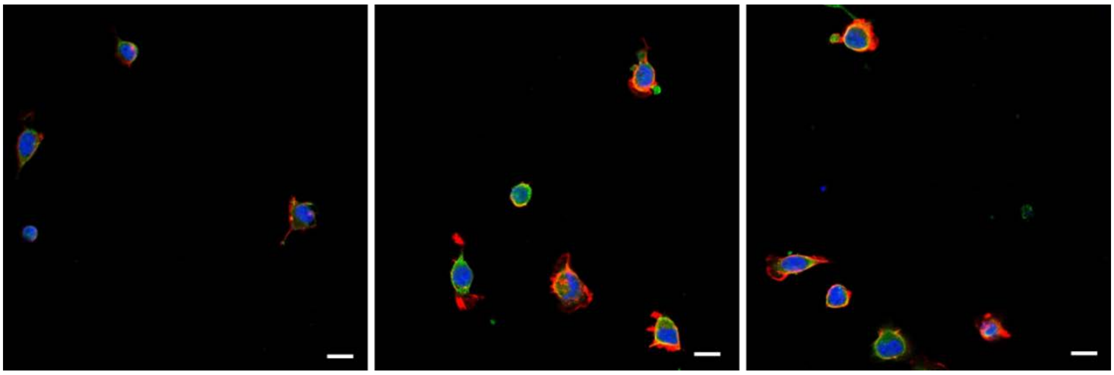

Supplementary Figure 64 | Confocal images of NG108-15 cells adhered on D-Pen-NP films functionalized with 40 mM D-Pen (8 hr) without addition of retinoic acid. (Red, actin; green, vinculin; blue, nucleus). Scale bars were 20μm.

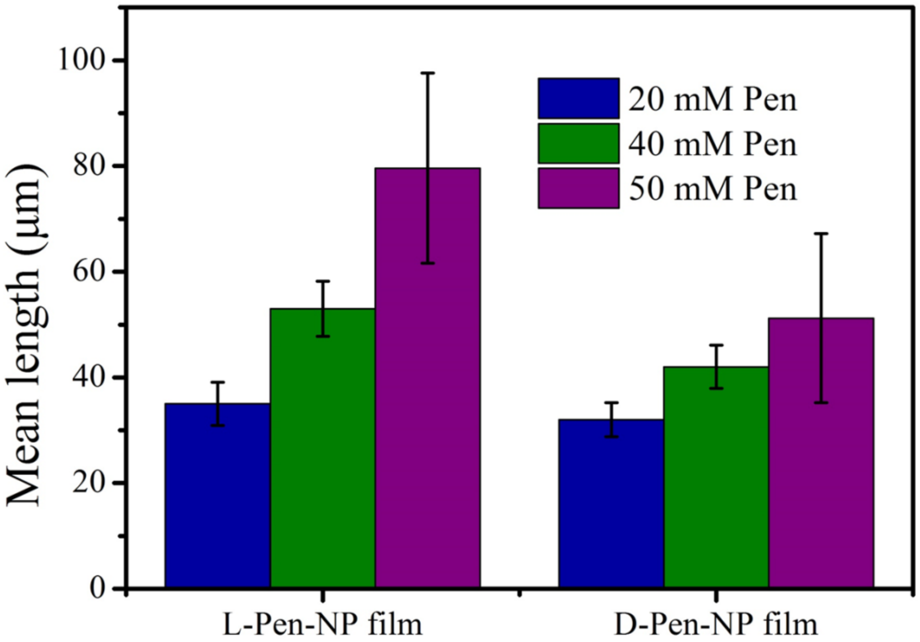

Supplementary Figure 65 | Mean lengths of neurites of NG108-15 cells differentiated on chiral films modified with different amounts of L/D-Pen with addition of RA. The error bars correspond to the standard error of the mean (n=6).

For the effects of the amount of L- or D-Pen on cell morphology (Supplementary Figure62), the average aspect ratio of cells adhered on L-Pen-NP film increased with the increase of amount of L-Pen. Cells on L-Pen(20 mM)-NP film exhibited an average aspect ratio of 2.7 ± 0.3, which was lower than that on L-Pen(40 mM, 50 mM)-NP film (3.4 ± 0.5, 5.3 ± 1.2 respectively).

As for cells adhered on D-Pen-NP films, although there was no obvious difference between the average aspect ratio of cells (about 1.5), the cell sticking area displayed smaller when the amounts of D-Pen increased (Supplementary Figure63-64, 50 mM D-Pen functionalized Au NP film in main text). With addition of RA, the average length of neurites showed increasing trend with the increase of D-Pen amounts (Supplementary Figure65).

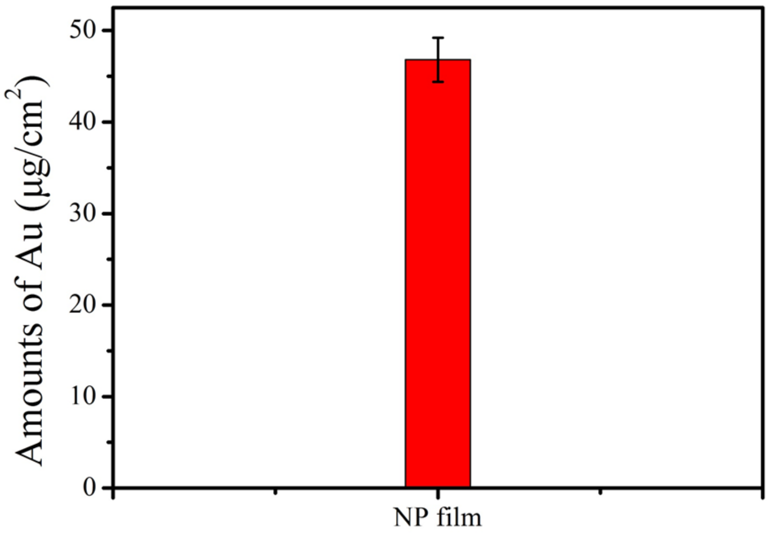

Supplementary Figure 66 | Amounts of Au element of Au NP film (without Pen modification and before protein adsorption). The amounts of N and S element were lower than the limit of detection. The error bars correspond to the standard error of the mean (n=3).

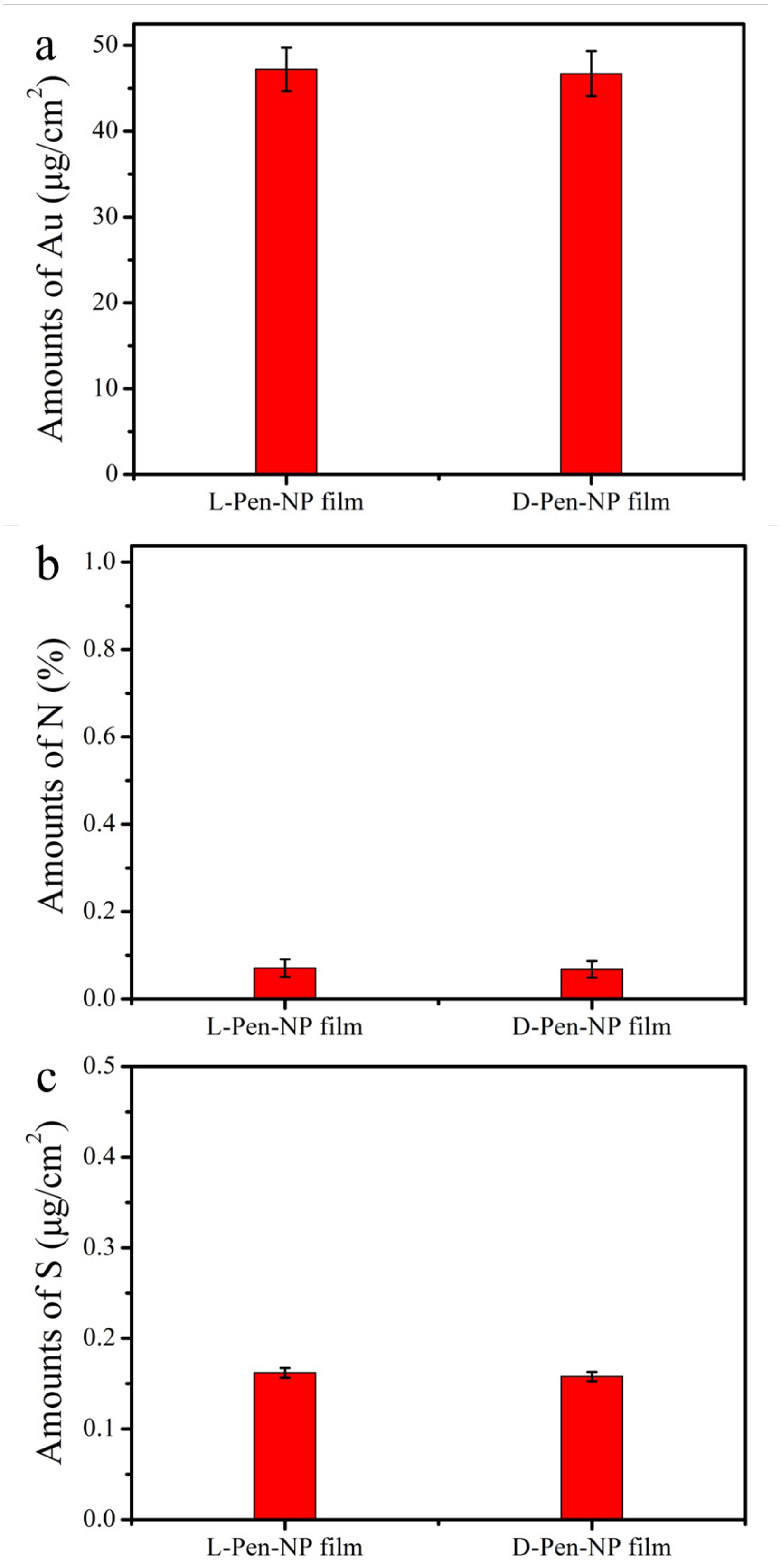

Supplementary Figure 67 | Amounts of (a) Au, (b) N and (c) S element of the L/D-Pen (50 mM) modified Au NP films before protein adsorption. The error bars correspond to the standard error of the mean (n=3).

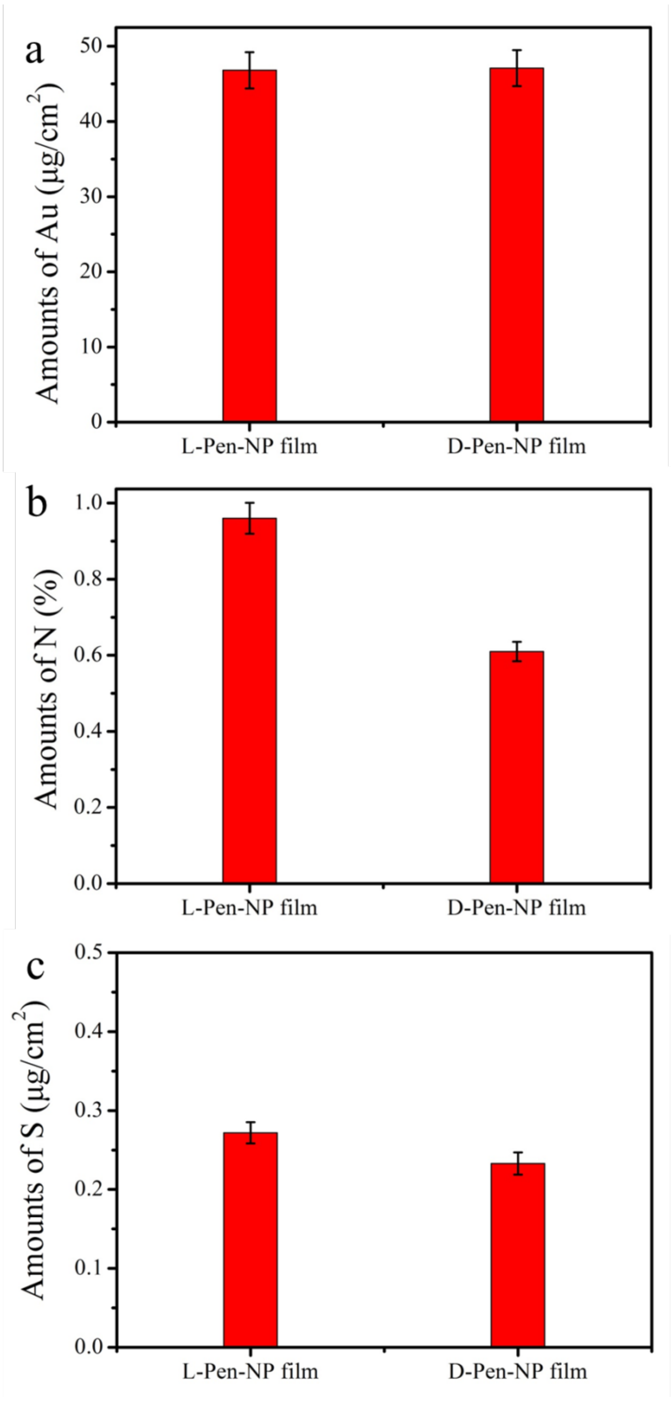

Supplementary Figure 68 | Amounts of (a) Au, (b) N and (c) S element of the L/D-Pen (50 mM) modified Au NP films after protein adsorption. The error bars correspond to the standard error of the mean (n=3).

Note: To measure the Au, N, and S elements respectively, the related fabricated Au NP films were scraped off from the glass substrates carefully and suspended in 50% ethanol for test. The S element was tested by ion chromatography (DIONEX

ICS-3000, USA)[3-4]. The Au element was tested by were determined by using an inductively coupled plasma optical emission spectrometer (ICP-OES, Thermo scientific iCAP 6300)[5-6]. N element was acquired by Elementar vario EL cube (Analysensysteme GmbH, Germany)[7-8].

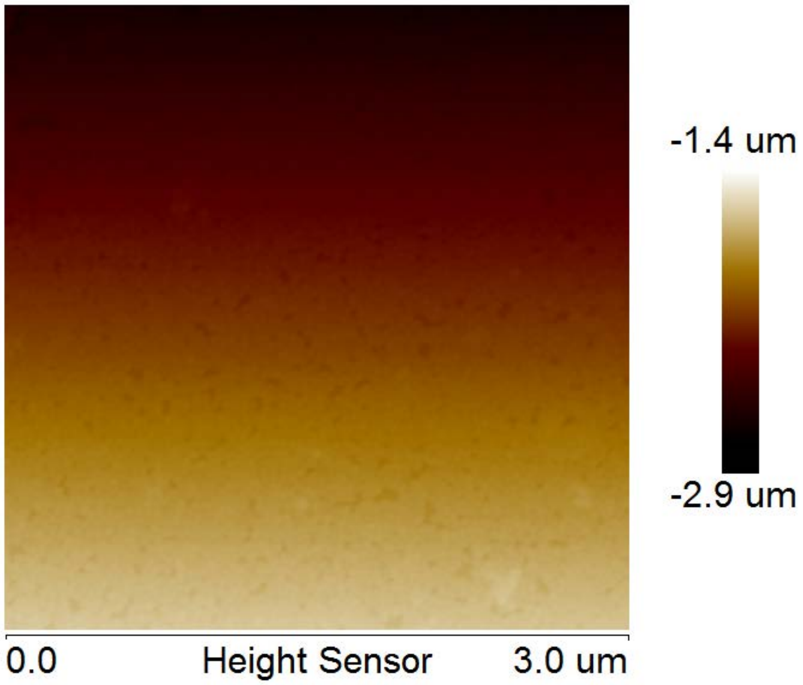

Supplementary Figure 69 | Original 2D image of Au NP film on glass substrate before flattening.

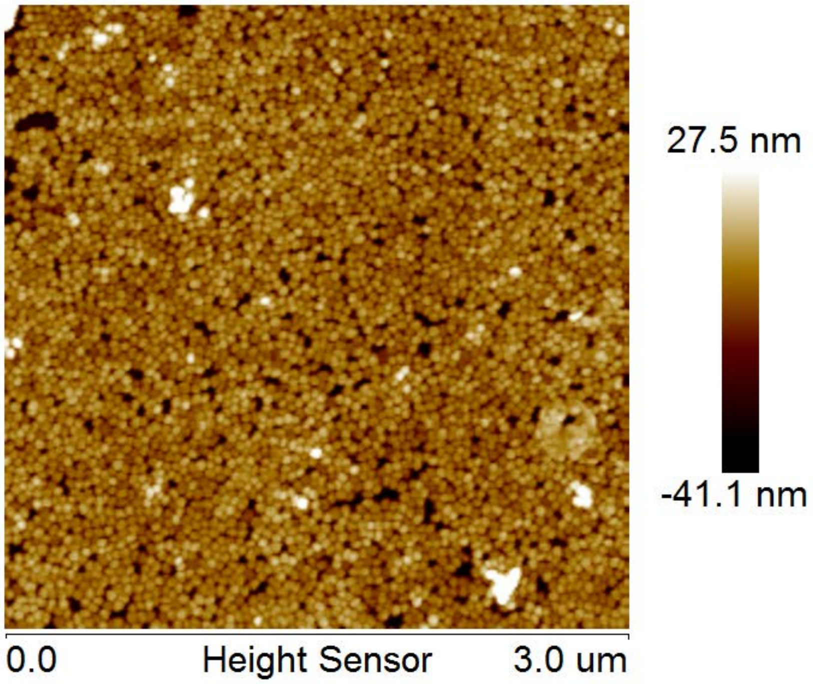

Supplementary Figure 70 | 2D image of Au NP film on glass substrate after flattening.

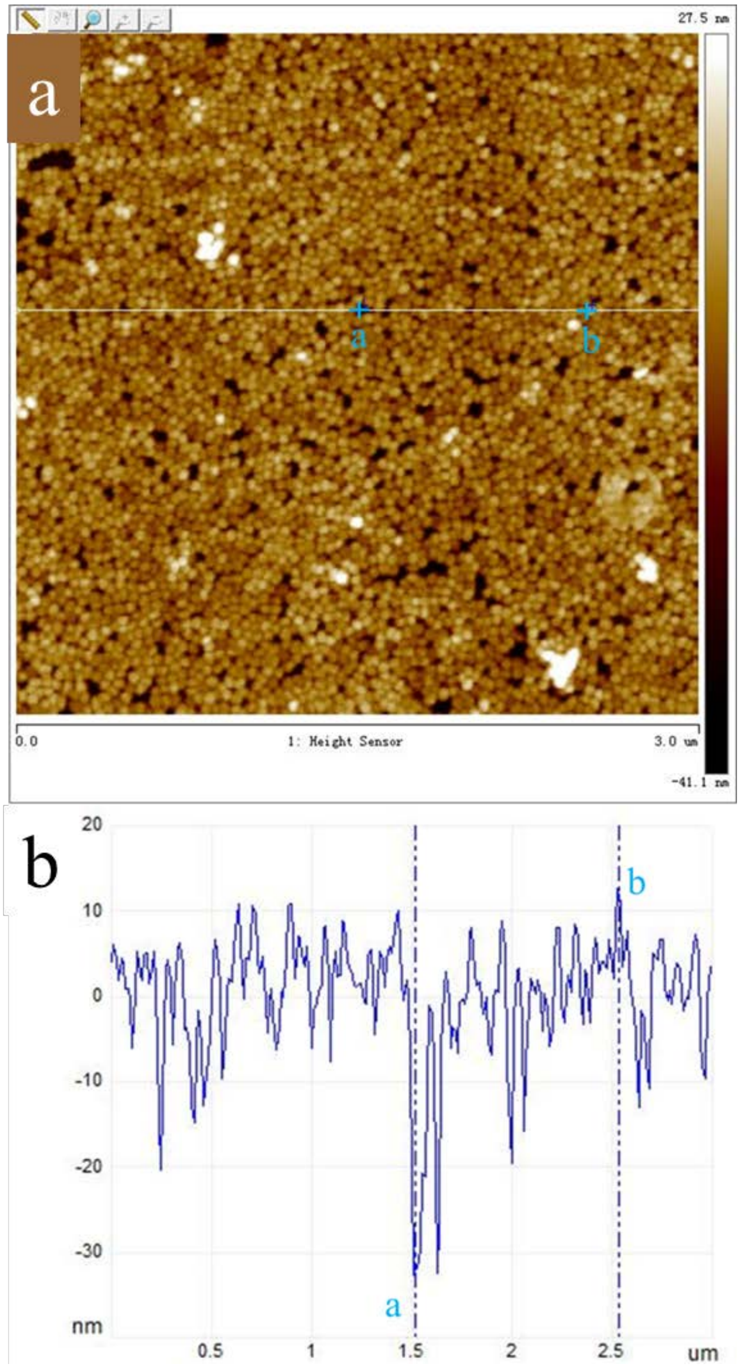

Supplementary Figure 71 | a) 2D image of Au NP film on glass substrate after flattening. b) Corresponding vertical distance.

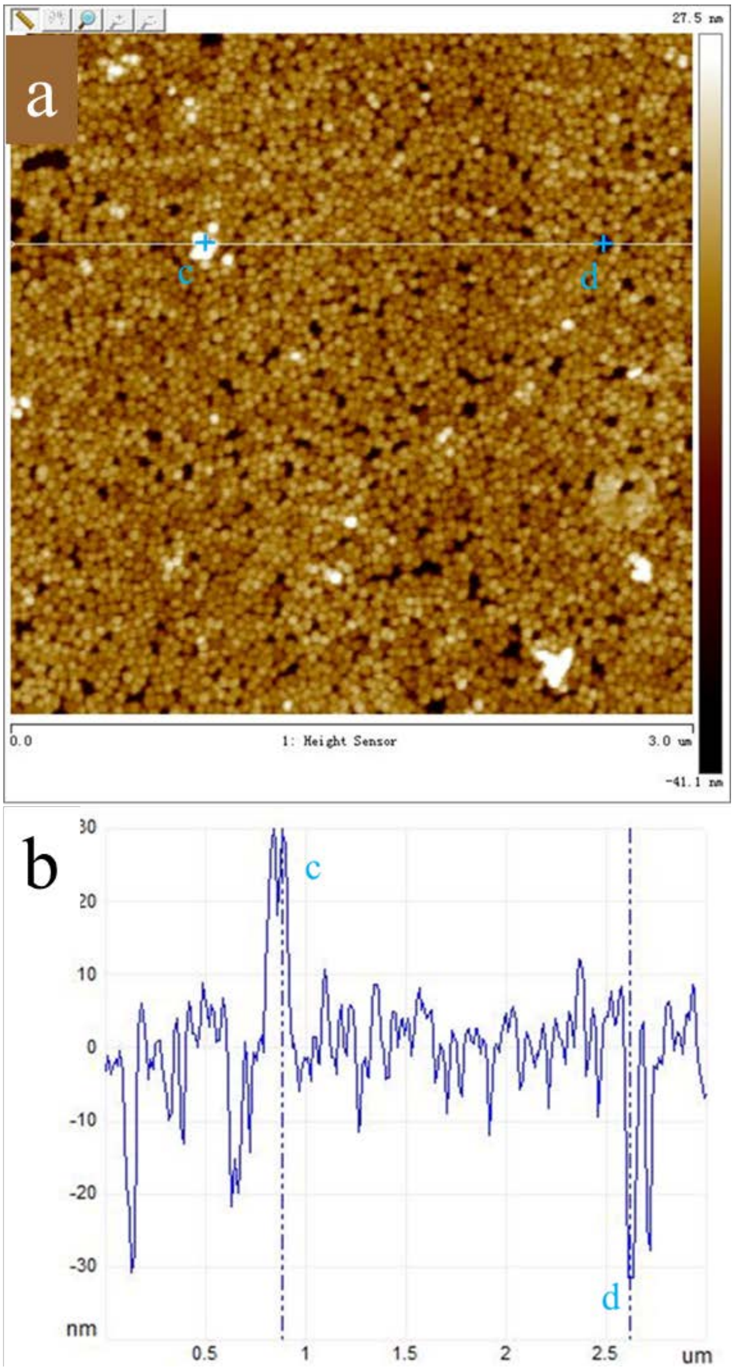

Supplementary Figure 72 | a) 2D image of Au NP film on glass substrate after flattening. b) Corresponding vertical distance.

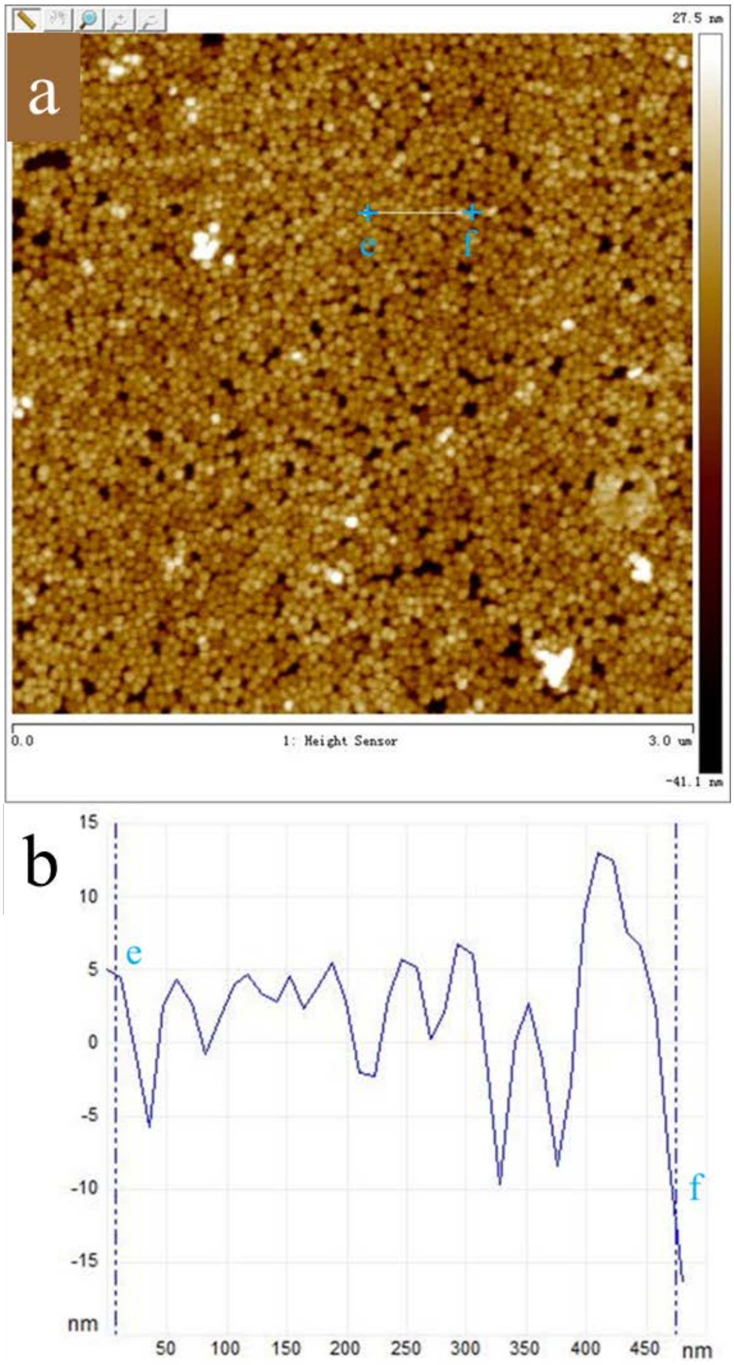

Supplementary Figure 73 | a) 2D image of Au NP film on glass substrate after flattening. b) Corresponding vertical distance.

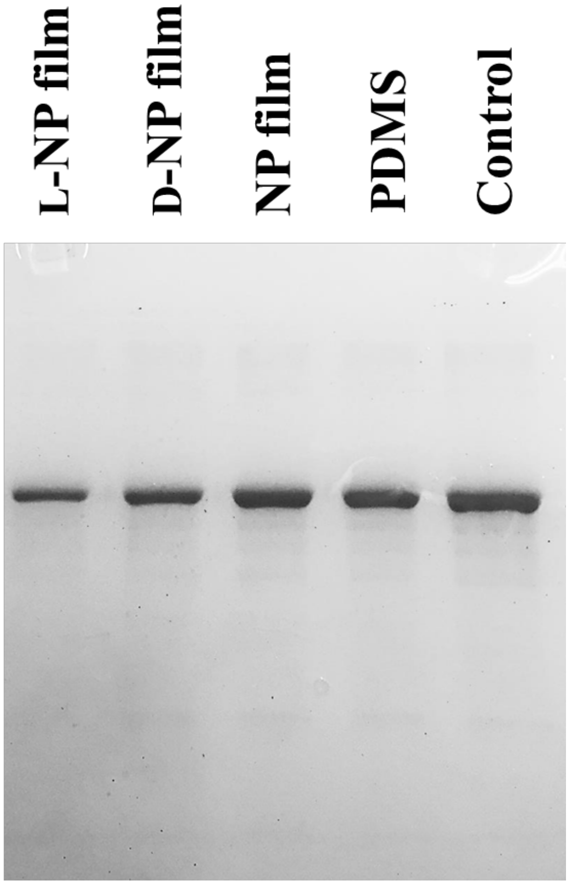

Supplementary Figure 74 | Expression of N-myc protein in differentiated NG108-15 cells.

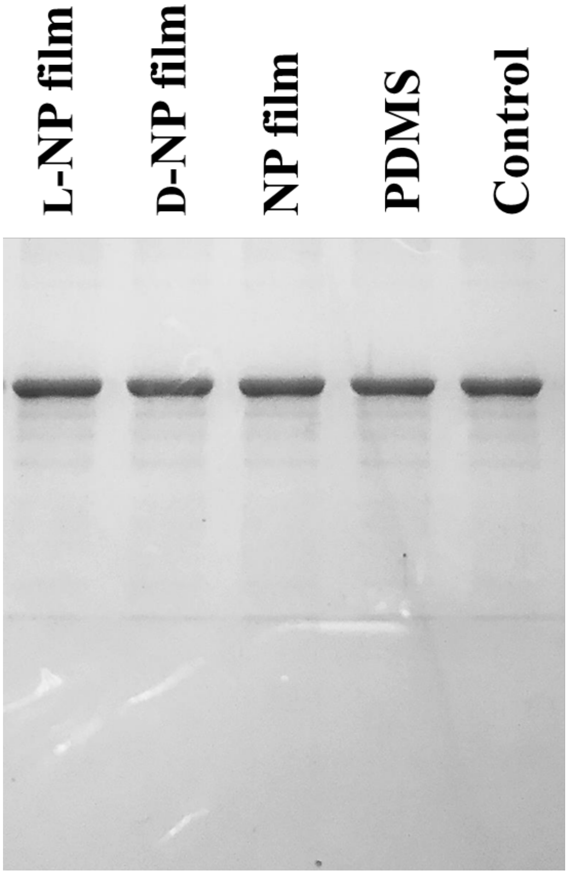

Supplementary Figure 75 | Expression of GAPDH protein (as a reference protein) in differentiated NG108-15 cells.

Supplementary References

(1) Zhao, X.; Xu, L.; Sun, M.; Ma, W.; Wu, X.; Kuang, H.; Wang, L.; Xu, C., Gold-Quantum Dot Core-Satellite Assemblies for Lighting Up MicroRNA In Vitro and In Vivo, *Small*, **12,** 4662-4668 (2016).

(2) Chen, H. Y.; Lin, M. H.; Wang, C. Y.; Chang, Y. M.; Gwo, S., Large-Scale Hot Spot Engineering for Quantitative SERS at the Single-Molecule Scale, *J. Am. Chem. Soc.* **137**, 13698-13705 (2015).

(3) Lee, C. Y.; Ho, K. L.; Lee, D. J.; Su, A.; Chang, J.-S., Electricity harvest from nitrate/sulfide-containing wastewaters using microbial fuel cell with autotrophic denitrifier, Pseudomonas sp. C27, *Int. J. Hydrogen Energ.* **37**, 15827-15832 (2012).

(4) Li, J.; Zhuang, G.; Huang, K.; Lin, Y.; Xu, C.; Yu, S., Characteristics and sources of air-borne particulate in Urumqi, China, the upstream area of Asia dust, *Atmos. Environ.*, **42**, 776-787 (2008).

(5) Sun, J.; Yang, X., Gold nanoclusters–Cu^{2+} ensemble-based fluorescence turn-on and real-time assay for acetylcholinesterase activity and inhibitor screening, *Biosens. Bioelectron.* **74**, 177-182 (2015).

(6) Sun, J.; Yang, F.; Yang, X., Synthesis of functionalized fluorescent gold nanoclusters for acid phosphatase sensing, *Nanoscale*, **7**, 16372-16380 (2015).

(7) Wang, K.; Li, Q.; Liu, B.; Cheng, B.; Ho, W.; Yu, J., Sulfur-doped g-C_3N_4 with enhanced photocatalytic CO_2-reduction performance, *Appl. Catal. B-Environ.* **176**, 44-52 (2015).

(8) Zhu, D.; Li, L.; Cai, J.; Jiang, M.; Qi, J.; Zhao, X., Nitrogen-doped porous carbons from bipyridine-based metal-organic frameworks: Electrocatalysis for oxygen reduction reaction and Pt-catalyst support for methanol electrooxidation, *Carbon*, **79**, 544-553 (2014).
